# CA-MuSiC: a Culture-Aware Multilingual Skill Cognition Model for MOOC review understanding

**DOI:** 10.3389/fpsyg.2026.1841361

**Published:** 2026-06-15

**Authors:** Guang Du, Biyuan Ma, Yan Liu

**Affiliations:** 1 School of English Studies, Zhejiang International Studies University, Hangzhou, Zhejiang, China; 2 Faculty of Education, Kunming City University, Kunming, Yunnan, China; 3 Beijing Institute of Economics and Management, Beijing, China

**Keywords:** aspect-based sentiment analysis, cross-lingual sentiment analysis, culture-aware learning, educational psychology, learning analytics, MOOC reviews, vocational skill cognition

## Abstract

**Introduction:**

Massive open online course (MOOC) reviews capture learners' emotional evaluations of course quality and perceived vocational value. Existing multilingual aspect-based sentiment analysis (ABSA) methods typically predict generic opinion structures but rarely account for context-conditioned differences in evaluative expression or link reviews to explicit skill perceptions.

**Methods:**

We propose CA-MuSiC, a Culture-Aware Multilingual Skill Cognition Model for MOOC review understanding. In this study, “culture-aware” is used in an operational sense: the model uses language, platform/source, and course discipline as observable cultural-context proxies, rather than claiming to measure learners' cultural identities directly. CA-MuSiC combines cultural-context adaptation, external skill grounding from the Course-Skill Atlas and O*NET, hybrid extractive-generative prediction, and teacher-ensemble pseudo-label bootstrapping.

**Results:**

Experiments on M-ABSA, EduRABSA, and an English-Chinese cross-lingual target-domain MOOC benchmark show that CA-MuSiC achieves the best results across TASD Micro-F1, ASQE F1, and Skill-grounded Sentiment Macro-F1, reaching 74.62, 61.45, and 69.88, respectively. Ablation studies indicate that skill grounding and pseudo-label bootstrapping are especially important for target-domain performance, whereas cultural-context adaptation improves cross-lingual robustness.

**Discussion:**

These findings contribute to educational psychology and learning analytics by modeling MOOC reviews as emotionally expressed learner evaluations of perceived skill development, rather than as mere satisfaction signals.

## 1 Introduction

Massive Open Online Courses (MOOCs) have become an important component of online higher education, providing learners with flexible access to vocationally relevant knowledge and skill-oriented learning opportunities. As MOOC ecosystems continue to expand across languages, regions, and educational cultures, large volumes of learner-generated reviews have become a valuable source of feedback for understanding how students perceive course quality, usefulness, and skill relevance (Yu et al., 2020, 2021; Onan, 2021). Compared with coarse-grained course ratings, review texts provide richer signals about whether a course is considered practical, transferable, employment-oriented, or sufficiently hands-on (Chen et al., 2022; Wang et al., 2021; Peng and Jiang, 2022). Therefore, automatically mining multilingual MOOC reviews is important not only for sentiment analysis but also for understanding how learners across different cultural contexts evaluate vocational skill development in online higher education.

Recent years have witnessed substantial progress in aspect-based sentiment analysis (ABSA), multilingual sentiment modeling, and educational review mining. On the one hand, general ABSA research has evolved from traditional extraction or classification pipelines to more unified and instruction-driven paradigms, including efficient hybrid generation, instruction-based ABSA, and large-scale multilingual benchmarking (Lv et al., 2023; Scaria et al., 2024; Wu et al., 2025). On the other hand, the education domain has gradually attracted increasing attention, with recent studies exploring transformer-based feedback analysis, deep opinion mining for course reviews, and fine-grained sentiment analysis in MOOC environments (Abdi et al., 2023; Id et al., 2024; Koufakou, 2024; Hua et al., 2025). However, existing studies still have several important limitations. First, most multilingual ABSA methods are designed for general domains rather than for educational review settings. Second, educational review studies often focus on overall satisfaction, topic detection, or broad feedback categorization, without explicitly modeling vocational skill cognition. Third, cross-cultural differences in emotional expression are typically treated as background variation rather than as a core factor shaping how learners perceive course-related skills (van Rijn and Larrouy-Maestri, 2023; Chen et al., 2024).

These observations suggest that multilingual MOOC review understanding in online higher education should not be framed as a simple sentiment classification problem. Instead, it should be formulated as a *culture-aware and skill-grounded educational interpretation task*. This task asks the model to jointly infer (*i*) the learner's emotional attitude toward a course-related experience, (*ii*) the aspect or category being evaluated, and (*iii*) the vocational skill cognition dimension that is being valued, questioned, or criticized. In this sense, learner reviews are treated as psychologically meaningful feedback about perceived learning value, rather than merely as positive or negative text.

We used the term *culture-aware* cautiously and operationally. Culture is not directly observed at the individual level in the public review data, and the model does not infer personal cultural identity. Instead, CA-MuSiC uses language, platform/source, and course discipline as observable cultural-context indicators. These indicators are imperfect but informative proxies for differences in communication norms, educational expectations, and evaluative framing. This operationalization is consistent with cross-cultural psychology, where emotional display rules, high- and low-context communication, cultural values, and self-construal are understood to influence how people express affective and evaluative meaning (Ekman and Friesen, 1969; Hall, 1976; Hofstede, 2001; Markus and Kitayama, 1991). Accordingly, our claim is not that the model fully measures culture, but that it explicitly models contextual variation that may otherwise be treated as noise.

The proposed task differs from conventional ABSA in both input and output. Standard ABSA typically maps a review text *t*_*i*_ to aspect–opinion–category–polarity tuples. The proposed educational interpretation task maps (*t*_*i*_, *l*_*i*_, *p*_*i*_, *d*_*i*_, *q*_*i*_), where *l*_*i*_, *p*_*i*_, *d*_*i*_, and *q*_*i*_ denote language, platform, discipline, and course metadata, to both ABSA-style opinion structures and skill-grounded sentiment labels. Thus, the novelty of the task formulation lies in reframing MOOC review analysis as learner emotion, learner evaluation, and skill perception modeling. The novelty of the model architecture lies in the specific CA-MuSiC design used to solve this task.

To address this issue, we propose CA-MuSiC, a Culture-Aware Multilingual Skill Cognition Model for MOOC review understanding. CA-MuSiC integrates four components into a unified model. First, it introduces a cultural-context adaptation module to separate shared educational meaning from context-specific expressive variation. Second, it incorporates skill grounding from the Course-Skill Atlas and O*NET, enabling MOOC reviews to be interpreted in terms of explicit vocational skill cognition dimensions rather than generic satisfaction alone (Sabet et al., 2024; Handel, 2016). Third, it uses a hybrid extractive–generative architecture with an mT5 decoder, enabling the model to handle both explicit span-based sentiment structures and implicit, compositionally complex educational reviews (Xue et al., 2021). Fourth, it adopts a three-teacher ensemble pseudo-label bootstrapping strategy to improve adaptation to the target domain under limited gold supervision.

The main contributions of this paper are summarized as follows:

- Task formulation. We define MOOC review understanding as a culture-aware and skill-grounded educational interpretation task, thereby extending conventional ABSA beyond generic polarity prediction to learner emotion, learner evaluation, and perceived skill-value analysis.
- Model architecture. We propose CA-MuSiC, a unified model that combines cultural-context adaptation, external skill grounding, hybrid extractive–generative prediction, and three-teacher ensemble pseudo-label bootstrapping.
- Empirical evaluation and interpretation. We conduct experiments on M-ABSA, EduRABSA, and an English–Chinese cross-lingual target-domain MOOC benchmark. The target-domain benchmark is therefore described as cross-lingual or bilingual rather than fully multilingual, while the broader training and evaluation setting still includes multilingual ABSA evidence.

The remainder of this paper is organized as follows. Section 2 reviews the related literature on multilingual ABSA, educational sentiment analysis, and skill-oriented review understanding. Section 3 presents the proposed CA-MuSiC model in detail. Section 4 introduces the datasets, baselines, experimental settings, and quantitative results. Section 5 concludes the paper and discusses future directions.

## 2 Related work

This section reviews prior research most relevant to our study from four perspectives: (*i*) general and multilingual ABSA, (*ii*) sentiment analysis and fine-grained opinion mining in educational reviews and MOOCs, (*iii*) skill-aware and knowledge-grounded educational review understanding, and (*iv*) culture-aware modeling in educational NLP. Taken together, these research streams underscore the need to understand a unified model of culture-aware and skill-grounded multilingual MOOC reviews understanding.

### 2.1 General and multilingual aspect-based sentiment analysis

Aspect-based sentiment analysis (ABSA) aims to identify fine-grained opinion structures by extracting sentiment-bearing aspects, opinions, and categories, along with their corresponding polarities. Compared with sentence-level sentiment classification, ABSA provides a more detailed view of *what* is being evaluated and *how* it is evaluated, and has therefore become a central paradigm for fine-grained opinion mining.

Recent ABSA research has gradually shifted from pipeline-style extraction and classification models toward more unified, end-to-end models. A systematic review by Zhang et al. (2023) and more recent surveys by Wankhade et al. (2024) and Zou et al. (2025) show that ABSA research has expanded rapidly in recent years. However, much of this progress remains concentrated on English-language and commercial-domain datasets, which may limit generalization to multilingual and educational review settings.

Regarding modeling paradigms, recent advances can be broadly grouped into three lines. The first line focuses on *generation-based or hybrid end-to-end models*. For example, EHG (Lv et al., 2023) improves the efficiency of end-to-end ABSA by jointly generating structural and semantic information, thereby alleviating the inefficiency of purely autoregressive generation. The second line emphasizes *instruction-based or unified task formulation*. InstructABSA (Scaria et al., 2024) demonstrates that instruction learning can effectively unify multiple ABSA subtasks and improve downstream performance. The third line focuses on *cross-lingual or multilingual transfer*. This direction has become increasingly important because most ABSA resources remain English-centric. M-ABSA (Wu et al., 2025) substantially expands multilingual ABSA benchmarking by providing a large-scale, multilingual dataset spanning multiple domains and languages, enabling more systematic evaluation of multilingual transfer and cross-lingual generalization.

Despite this progress, two limitations remain particularly relevant to our study. First, most existing ABSA methods are not designed for educational review texts, whose discourse is often longer, more implicit, and more pedagogically structured than that of standard product or service reviews. Second, even multilingual ABSA methods usually treat language variation primarily as a transfer challenge, rather than explicitly modeling culture-conditioned expression bias. These limitations make it difficult to directly apply general ABSA methods to the understanding of multilingual MOOC reviews.

### 2.2 Educational sentiment analysis and MOOC review mining

Sentiment analysis in educational settings has attracted growing attention because learner-generated feedback can provide valuable evidence about teaching quality, course experience, and learning outcomes. Compared with commercial reviews, educational feedback often provides richer pedagogical signals, such as perceived difficulty, instructional clarity, assessment fairness, and practical usefulness. However, educational sentiment analysis has historically relied more on coarse-grained document or sentence-level polarity classification than on fine-grained structured opinion mining.

Recent studies have begun to apply transformer-based and domain-adapted models to educational feedback analysis. For example, recent research has examined transformer-based classification of student evaluation feedback and online course reviews, suggesting that modern pre-trained models can outperform traditional lexicon-based or shallow machine learning approaches (Abdi et al., 2023; Id et al., 2024; Pilicita-Garrido and Barra, 2025). Simultaneously, research on MOOC review mining has increasingly emphasized fine-grained sentiment and topic analyses in online course settings (Onan, 2021; Chen et al., 2022; Peng and Jiang, 2022; Koufakou, 2024). These studies indicate that MOOC review corpora contain diverse sentiment-bearing structures and support more detailed analysis than simple overall ratings.

A particularly important recent development is the release of EduRABSA (Hua et al., 2025), which, to the best of our knowledge, is the first publicly annotated ABSA dataset dedicated to education reviews. EduRABSA is significant because it provides a structured benchmark for aspect–opinion–category–sentiment analysis in educational feedback, including implicit aspect and implicit opinion cases. Its release substantially lowers the barrier to educational ABSA research and enables evaluation of fine-grained opinion-mining models in a more realistic academic domain.

Nevertheless, existing research on educational sentiment analysis still has at least three shortcomings for our target problem. First, many studies remain focused on English-only or single-platform settings, limiting their usefulness in multilingual MOOC environments. Second, even when educational reviews are analyzed at a fine-grained level, most studies still emphasize general educational satisfaction rather than explicit vocational skill cognition. Third, public educational ABSA resources remain limited in both scale and diversity, especially in multilingual course-review settings. These limitations underscore the need to combine educational review mining with multilingual transfer and target-domain adaptation.

### 2.3 Skill-aware and knowledge-grounded educational review understanding

Although sentiment analysis can indicate whether learners evaluate a course positively or negatively, this information alone is often insufficient for vocationally oriented higher education. In many online learning scenarios, the more important question is whether a course is perceived as practical, transferable, employment-relevant, or sufficiently hands-on. Such judgments are not merely sentiment labels; they reflect *skill cognition*. However, explicit modeling of skill cognition remains underexplored in the existing review-mining and ABSA literature.

The six skill cognition categories used in this study are grounded in educational psychology, vocational education, and labor-market signaling literature. Practical utility corresponds to perceived usefulness and real-world applicability (Davis, 1989). Employability relevance reflects the perceived connection between learning and career adaptability or job readiness (Fugate et al., 2004). Skill acquisition concerns the development of cognitive skills, in which learners move from declarative understanding toward proceduralized competence (Anderson, 1982). Transferability follows training-transfer research, which emphasizes whether learned knowledge generalizes to new tasks and contexts (Baldwin and Ford, 1988). Practice adequacy is driven by experiential learning, in which concrete experience and active experimentation support learning outcomes (Kolb, 1984). Credential value reflects the signaling function of certificates and educational credentials in labor-market interpretation (Spence, 1973). These constructs justify the six-category space as a theoretically motivated coding scheme rather than a purely data-driven label inventory.

One promising direction is to incorporate external knowledge into text understanding. In general NLP and recommendation settings, knowledge grounding has been shown to improve interpretability and task alignment. In the educational domain, Course-Skill Atlas (Sabet et al., 2024) is a particularly valuable resource that links higher-education course descriptions to labor-market-oriented skill profiles. Similarly, O*NET offers a standardized taxonomy of occupational knowledge, skills, and work activities, and its strengths and limitations as an occupational information resource have been systematically discussed in prior research (Handel, 2016; National Research Council, 2010). Together, these resources provide a principled basis for grounding course reviews in explicit vocational skill structures rather than relying solely on free-form semantic correlations.

However, existing sentiment analysis and ABSA studies have rarely integrated such external occupational knowledge into the understanding of multilingual educational reviews. Most models still infer aspect importance directly from textual co-occurrence patterns, which may be insufficient when skill-related signals are implicit, weakly expressed, or semantically indirect. Therefore, explicit skill grounding is necessary to move from generic sentiment recognition to interpretable vocational skill cognition analysis in MOOC reviews.

### 2.4 Culture-aware modeling in educational NLP

Culture has long been recognized as a key factor in education, shaping how learners interpret pedagogical practices, communicate emotions, and evaluate learning experiences. However, in most computational sentiment analysis pipelines, culture is either ignored or reduced to a language label. This simplification can be problematic because language and cultural variations are related but not identical.

Students from different educational cultures may differ not only in lexical choice but also in the degree of directness, politeness, emphasis, and evaluative framing in reviews.

Cross-cultural psychology provides a stronger foundation for this argument. Display-rule theory suggests that cultures differ in norms for expressing emotion (Ekman and Friesen, 1969); Hall's high- vs. low-context communication model explains why some evaluative meanings may be expressed directly whereas others remain implicit (Hall, 1976); Hofstede's research highlights cultural value dimensions that can shape institutional expectations (Hofstede, 2001); and Markus and Kitayama's self-construal theory shows how cognition, emotion, and motivation can vary with culturally shaped views of the self (Markus and Kitayama, 1991). Recent empirical studies also show that affective expression and emotion regulation can vary across cultural contexts (van Rijn and Larrouy-Maestri, 2023; Chen et al., 2024).

Simultaneously, multilingual ABSA benchmarks such as M-ABSA (Wu et al., 2025) enable more systematic examination of cross-lingual generalization. However, explicit cultural-context representation learning remains underexplored in educational review understanding. Most existing methods treat multilingual transfer as a pure representation-alignment problem, without distinguishing shared educational meaning from context-specific expression patterns.

For multilingual MOOC reviews, this distinction is essential. A model that ignores culture may conflate pedagogically meaningful differences with noise, or fail to generalize across platforms where students express similar judgments in culturally distinct ways. Therefore, a more suitable approach should explicitly model both shared semantic structure and culture-specific expressive variation.

### 2.5 Summary and research gap

In summary, prior research has made significant progress in multilingual ABSA, educational sentiment analysis, and fine-grained review mining. General ABSA research has produced increasingly powerful end-to-end, instruction-based, and multilingual methods. Educational NLP has begun to shift from coarse sentiment prediction to more structured review understanding, and recent publicly available resources, such as EduRABSA, have significantly improved the feasibility of educational ABSA research. Meanwhile, external skill resources, such as the Course-Skill Atlas and O*NET, create new opportunities to ground educational text analysis in interpretable vocational knowledge.

However, no existing line of work fully addresses the problem examined in this study. Specifically, current approaches do not jointly model (*i*) multilingual and cross-cultural educational review understanding, (*ii*) fine-grained aspect-level sentiment reasoning, and (*iii*) explicit vocational skill cognition grounding. This gap motivates the proposed CA-MuSiC model, which integrates cultural-context adaptation, external skill grounding, hybrid extractive–generative prediction, and teacher-ensemble target-domain adaptation into a unified solution for MOOC review understanding. Formally, conventional ABSA predicts {(*a, o, c, s*)} from text, whereas the proposed task predicts {(*a, o, c, s*), **y**^cat^, **y**^pol^} from text, educational, and cultural-context metadata. This expanded output space is what makes the task educationally interpretable rather than only sentiment-classificatory.

**Figure 1:**
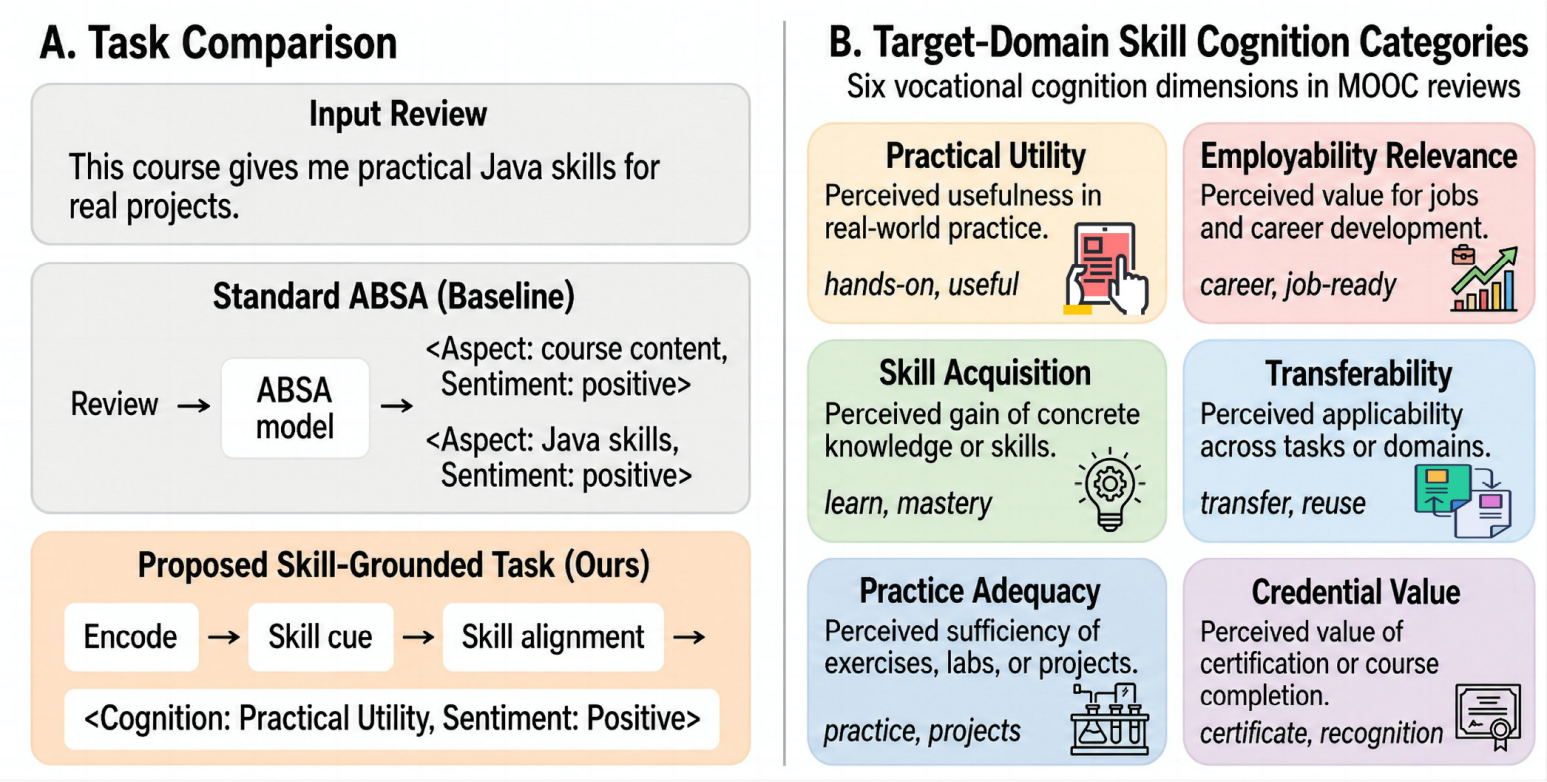
Comparison between standard ABSA and the proposed skill-grounded sentiment task, including the six target-domain skill cognition categories. **(A)** illustrates that conventional ABSA can identify aspects and sentiment polarity, but does not explicitly capture vocational skill cognition. Conversely, the proposed task further encodes the review text, identifies skill-related cues, aligns them with external skill priors, and predicts skill-grounded sentiment across predefined cognitive dimensions. **(B)** summarizes the six target-domain skill-cognition categories used in this study, namely practical utility, employability relevance, skill acquisition, transferability, practice adequacy, and credential value.

To further illustrate the distinction between conventional ABSA and the task examined in this paper, Figure 1 presents a schematic comparison.

## 3 Methodology

In this study, we propose CA-MuSiC Culture-Aware Multilingual Skill Cognition Model), a culture-adaptive, skill-grounded, hybrid extractive–generative model for MOOC review understanding. To make the method accessible to readers in education and psychology, we first describe the model in plain language. CA-MuSiC reads a learner review, uses available context indicators such as language, platform, and discipline to reduce expression bias, links review content to external skill knowledge, and then predicts both conventional ABSA outputs and skill-grounded sentiment labels. The equations below specify how these steps are implemented for reproducibility.

CA-MuSiC is designed to address three coupled challenges. First, learners may express similar emotions and evaluations with varying degrees of directness, politeness, or implicitness across languages and platforms. Second, vocationally oriented course reviews should not be reduced to generic satisfaction signals, because learners often evaluate whether a course is useful, career-relevant, transferable, sufficiently practical, or credential-worthy. Third, the target-domain MOOC review corpus has limited gold annotations, making direct supervised learning insufficient. CA-MuSiC therefore learns progressively from multilingual ABSA data, education-domain ABSA data, and English–Chinese target-domain MOOC reviews. The full mathematical formulation of CA-MuSiC is provided in Equations 1–68

### 3.1 Problem definition

Let $D={\left\{\left(x_{i},Y_{i}\right)\right\}}_{i=1}^{N}$ denote the overall corpus used for training and evaluation, where *x*_*i*_ is the input review instance and $Y_{i}$ is the corresponding supervision signal. Because CA-MuSiC is trained on multiple public datasets with heterogeneous label schemas, we first define a unified input–output representation that serves as a superset across training stages.

Throughout this section, $Y_{i}$ denotes gold-standard supervision when it is available, whereas ${\hat{Y}}_{i}$ denotes the prediction from CA-MuSiC. This distinction is important because not all datasets provide every label type.

For the *i*-th review, the input instance is defined as


$$\begin{array}{l} x_{i}=\left\{t_{i},l_{i},p_{i},d_{i},q_{i}\right\}, \end{array}$$ (1)

where *t*_*i*_ = [*w*_*i*1_, *w*_*i*2_, …, *w*_*i*_*n*__*i*__] is the raw review text, $l_{i}\in L$ is the language label, $p_{i}\in P$ is the platform/source indicator, $d_{i}\in D^{\text{dom}}$ is the discipline or domain label when available, and *q*_*i*_ denotes the associated course metadata text, such as the title, description, and auxiliary course information.

To unify different annotation formats, we represent the fine-grained sentiment structure as a set of structured units:


$$\begin{array}{l} Y_{i}^{\text{tuple}}={\left\{\left(a_{ij},o_{ij},c_{ij},s_{ij}\right)\right\}}_{j=1}^{M_{i}}, \end{array}$$ (2)

where *M*_*i*_ is the number of sentiment units in review *i*, *a*_*ij*_ denotes the aspect term (or implicit aspect), *o*_*ij*_ denotes the opinion term (or implicit opinion), *c*_*ij*_ denotes the category label, and *s*_*ij*_ denotes the sentiment polarity. The polarity label space is defined as


$$\begin{array}{l} S=\left\{\text{positive},\text{neutral},\text{negative}\right\}. \end{array}$$ (3)

In addition to tuple-level supervision, some datasets provide sentence-level sentiment labels, denoted by


$$\begin{array}{l} y_{i}^{\text{sent}}\in S. \end{array}$$ (4)

For the target-domain task, category semantics are further projected into a predefined vocational skill cognition space:


$$\begin{array}{l} C^{\text{skill}}=\left\{c^{\left(1\right)},c^{\left(2\right)},\ldots ,c^{\left(K\right)}\right\}, \end{array}$$ (5)

where *K* denotes the number of target-domain skill categories. In this study, *K* = 6, and the six skill cognition categories are defined as


$$\begin{array}{c} C^{\text{skill}}=\\ \left\{\begin{array}{l} \text{practical\_utility},\text{employability\_relevance},\\ \text{skill\_acquisition},\text{transferability},\\ \text{practice\_adequacy},\text{credential\_value} \end{array}\right\}. \end{array}$$ (6)

The six skill cognition categories were developed through a three-step procedure. First, we derived candidate dimensions from the educational psychology and vocational education literature cited in Section 2.3. Second, we aligned these dimensions with the Course-Skill Atlas and O*NET descriptors to ensure that each category was grounded in external skill knowledge. Third, we refined the definitions during the pilot annotation round described in Section 4.2.4. The categories are *multi-label and potentially overlapping*; for example, a review may praise both practical utility and employability relevance. They are not intended to form a strict hierarchy. Table 1 provides definitions and illustrative examples.

**Table 1:** Conceptual definition of the six target-domain skill cognition categories.

Category	Theoretical basis	Definition	Illustrative review example
Practical utility	Perceived usefulness	The learner perceives the course as useful for real-world tasks or immediate application.	“The Excel cases are useful for my daily accounting work.”
Employability relevance	Employability and career adaptability	The learner perceives the course as helpful for jobs, career development, or job readiness.	“The portfolio project helped me prepare for data analyst roles.”
Skill acquisition	Cognitive skill development	The learner reports gaining concrete knowledge, techniques, or procedural competence.	“I finally learned how to build a neural network from scratch.”
Transferability	Training transfer	The learner perceives that knowledge or skills can be reused across tasks, domains, or settings.	“The statistical methods transfer to my biology research.”
Practice adequacy	Experiential learning	The learner evaluates whether exercises, labs, cases, or projects are sufficient for practice.	“The course needs more hands-on coding assignments.”
Credential value	Labor-market signaling	The learner evaluates the value of certificates, completion records, or formal recognition.	“The certificate is useful for my CV.”

For skill-grounded prediction, we further define two target-domain supervision variables:


$$\begin{array}{l} y_{ik}^{\text{cat}}\in \left\{0,1\right\},\;y_{ik}^{\text{pol}}\in S, \end{array}$$ (7)

where $y_{ik}^{\text{cat}}$ indicates whether the *k*-th skill category is activated in review *i*, and $y_{ik}^{\text{pol}}$ denotes its corresponding polarity when the category is active.

Accordingly, the unified supervision space can be written as


$$\begin{array}{l} Y_{i}=\left\{Y_{i}^{\text{tuple}},y_{i}^{\text{sent}},{\text{y}}_{i}^{\text{cat}},{\text{y}}_{i}^{\text{pol}}\right\}, \end{array}$$ (8)

where ${\text{y}}_{i}^{\text{cat}}=\left[y_{i1}^{\text{cat}},\ldots ,y_{iK}^{\text{cat}}\right]$ and ${\text{y}}_{i}^{\text{pol}}=\left[y_{i1}^{\text{pol}},\ldots ,y_{iK}^{\text{pol}}\right]$ are available only for the target-domain task. In practice, different datasets supervise different subsets of $Y_{i}$.

Given an input review *x*_*i*_, the model aims to estimate


$$\begin{array}{l} {\hat{Y}}_{i}=f_{\theta }\left(x_{i}\right), \end{array}$$ (9)

where *f*_θ_(·) denotes CA-MuSiC parameterized by θ. Depending on the supervision available at each training stage, the prediction ${\hat{Y}}_{i}$ may include (*i*) sentence-level sentiment prediction, (*ii*) aspect and opinion extraction, (*iii*) category-level polarity prediction, and (*iv*) target-domain skill-grounded sentiment inference.

### 3.2 Overall architecture

**Figure 2:**
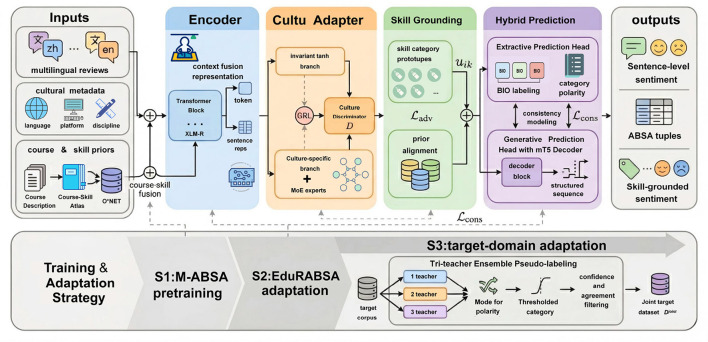
Overview of the proposed CA-MuSiC model. Given multilingual MOOC reviews, cultural metadata, and course-level skill priors, CA-MuSiC first encodes the input using a multilingual encoder to obtain contextualized token- and sentence-level representations. A culture adaptation module then disentangles culture-invariant and culture-specific features through adversarial cultural discrimination and mixture-of-experts modeling. Based on the culture-adapted representations and external course-skill priors, the skill grounding module aligns review semantics with vocational skill categories. The resulting skill-aware representations are then fed into a hybrid prediction module comprising an extractive prediction head and an mT5-based generative prediction head, with consistency modeling to promote coherent predictions across the two branches. The model produces three types of outputs, namely sentence-level sentiment, ABSA tuples, and skill-grounded sentiment. At the bottom, a three-stage training and adaptation strategy is illustrated, comprising multilingual ABSA pre-training, education-domain adaptation, and target-domain adaptation with tri-teacher ensemble pseudo-labeling, majority-vote polarity aggregation, category activation thresholding, and confidence/agreement filtering.

Figure 2 presents an overview of the proposed CA-MuSiC model. Given multilingual MOOC reviews, cultural metadata, and course-level skill priors, CA-MuSiC first maps heterogeneous inputs into a unified representation space and then encodes them using a multilingual semantic encoder to produce contextualized token- and sentence-level representations. A culture adaptation module subsequently disentangles culture-invariant semantics from culture-specific expression bias using adversarial cultural discrimination and a mixture-of-experts model. Based on the culture-adapted representations and external course-skill priors, a skill grounding module aligns review semantics with predefined vocational skill cognition categories. The resulting skill-aware representations are then fed into a hybrid prediction module consisting of an extractive branch and an mT5-based generative branch, with consistency regularization promoting coherent predictions across the two heads. Finally, the model is trained using a three-stage adaptation strategy that includes multilingual ABSA pre-training, education-domain adaptation, and target-domain adaptation with a three-teacher ensemble pseudo-labeling.

CA-MuSiC consists of five core modules:

1. Input Representation Layer, which transforms multilingual reviews, cultural context, and course metadata into a unified embedding space;
2. Multilingual Semantic Encoding Layer, which encodes reviews into contextualized token-level and sentence-level representations;
3. Culture Adaptation Layer, which disentangles culture-invariant and culture-specific semantics;
4. Skill Grounding Layer, which aligns review semantics with external skill priors derived from Course-Skill Atlas and O*NET;
5. Hybrid Extractive–Generative Prediction Layer, which jointly performs span extraction, category-level sentiment reasoning, and structured sequence generation.

The full inference process can be summarized as


$$\begin{array}{l} {\hat{Y}}_{i}=f_{\theta }^{\text{pred}}(f_{\theta }^{\text{skill}}(f_{\theta }^{\text{cult}}(f_{\theta }^{\text{enc}}(f_{\theta }^{\text{in}}\left(x_{i}\right))). \end{array}$$ (10)

This modular decomposition is important because each component addresses a distinct challenge. The input representation layer integrates heterogeneous educational signals, the multilingual encoder captures shared semantics across languages (Conneau et al., 2020), the culture adaptation module accounts for cross-cultural expression bias (van Rijn and Larrouy-Maestri, 2023; Chen et al., 2024); the skill grounding layer injects vocationally relevant external knowledge (Sabet et al., 2024; National Research Council, 2010), and the hybrid predictor improves structured sentiment reasoning under both explicit and implicit review conditions (Lv et al., 2023; Scaria et al., 2024; Xu et al., 2024).

### 3.3 Input representation layer

The input representation layer transforms heterogeneous information into a unified vector space. For each review, CA-MuSiC constructs three complementary inputs: textual representation, cultural context representation, and course-skill prior representation.

#### 3.3.1 Textual tokenization and embedding

Given the review text *t*_*i*_ = [*w*_*i*1_, *w*_*i*2_, …, *w*_*i*_*n*__*i*__], we first apply a shared multilingual tokenizer to obtain subword tokens:


$$\begin{array}{l} {\tilde{t}}_{i}=\left[{\tilde{w}}_{i1},{\tilde{w}}_{i2},\ldots ,{\tilde{w}}_{i{ñ}_{i}}\right], \end{array}$$ (11)

where ñ_*i*_ is the subword sequence length after tokenization.

Each subword token is then mapped to an embedding vector:


$$\begin{array}{l} {\text{e}}_{ij}^{\text{tok}}={\text{E}}^{\text{tok}}\left({\tilde{w}}_{ij}\right), \end{array}$$ (12)

where **E**^tok^(·) denotes the token embedding lookup function.

#### 3.3.2 Cultural context embedding

To model observable cultural-context heterogeneity without claiming to infer individual cultural identity, we encode language identity, source platform, and discipline/domain label. Let ${\text{e}}_{i}^{\text{lang}}$, ${\text{e}}_{i}^{\text{plat}}$, and ${\text{e}}_{i}^{\text{dom}}$ denote the corresponding trainable embeddings:


$$\begin{array}{l} {\text{e}}_{i}^{\text{lang}}={\text{E}}^{\text{lang}}\left(l_{i}\right),\;{\text{e}}_{i}^{\text{plat}}={\text{E}}^{\text{plat}}\left(p_{i}\right),\;{\text{e}}_{i}^{\text{dom}}={\text{E}}^{\text{dom}}\left(d_{i}\right), \end{array}$$ (13)

where *d*_*i*_ is the course discipline label when available.

The cultural context representation is then defined as


$$\begin{array}{l} {\text{c}}_{i}={\text{MLP}}_{c}\left(\left[{\text{e}}_{i}^{\text{lang}};{\text{e}}_{i}^{\text{plat}};{\text{e}}_{i}^{\text{dom}}\right]\right), \end{array}$$ (14)

where [·;·] denotes vector concatenation.

#### 3.3.3 Course-skill prior representation

Let *q*_*i*_ denote the metadata text associated with review *i*, including the course title, description, and auxiliary course information. We encode the metadata text using the same multilingual encoder backbone and obtain a pooled representation:


$$\begin{array}{l} {\text{g}}_{i}^{\text{meta}}=\text{Pool}(\text{Encoder}\left(q_{i}\right)). \end{array}$$ (15)

To support explicit skill grounding, we construct a set of external skill prototypes:


$$\begin{array}{l} R^{\text{skill}}=\left\{{\text{r}}_{1}^{\text{skill}},{\text{r}}_{2}^{\text{skill}},\ldots ,{\text{r}}_{K}^{\text{skill}}\right\}, \end{array}$$ (16)

where each ${\text{r}}_{k}^{\text{skill}}$ is derived from Course-Skill Atlas and O*NET resources and corresponds to one target-domain skill cognition category.

We then compute metadata-conditioned prior weights:


$$\begin{array}{l} \alpha_{ik}^{\text{meta}}=\frac{\exp({\left({\text{W}}_{q}{\text{g}}_{i}^{\text{meta}}\right)}^{⊤}\left({\text{W}}_{r}{\text{r}}_{k}^{\text{skill}}\right))}{\sum_{k^{\prime }=1}^{K}\exp({\left({\text{W}}_{q}{\text{g}}_{i}^{\text{meta}}\right)}^{⊤}\left({\text{W}}_{r}{\text{r}}_{k^{\prime }}^{\text{skill}}\right))}. \end{array}$$ (17)

The metadata-conditioned skill prior is


$$\begin{array}{l} {\tilde{\text{r}}}_{i}^{\text{meta}}=\overset{K}{\underset{k=1}{\sum }}\alpha_{ik}^{\text{meta}}{\text{r}}_{k}^{\text{skill}}. \end{array}$$ (18)

### 3.4 Multilingual semantic encoding layer

After obtaining textual and contextual inputs, we use a multilingual encoder to derive contextualized semantic representations. Specifically, CA-MuSiC adopts XLM-RoBERTa as the shared encoder because it provides strong multilingual generalization across diverse languages (Conneau et al., 2020).

For each subword token, we form the encoder input as


$$\begin{array}{l} {\text{x}}_{ij}^{\left(0\right)}={\text{e}}_{ij}^{\text{tok}}+{\text{e}}_{ij}^{\text{pos}}+{\text{W}}_{c}^{\left(0\right)}{\text{c}}_{i}, \end{array}$$ (19)

where ${\text{e}}_{ij}^{\text{pos}}$ is the positional embedding and ${\text{W}}_{c}^{\left(0\right)}{\text{c}}_{i}$ injects the cultural context into the token representation.

The encoder output is


$$\begin{array}{l} {\text{H}}_{i}=\left[{\text{h}}_{i1},{\text{h}}_{i2},\ldots ,{\text{h}}_{i{ñ}_{i}}\right]=\text{XLMR}\left({\text{x}}_{i1}^{\left(0\right)},\ldots ,{\text{x}}_{i{ñ}_{i}}^{\left(0\right)}\right), \end{array}$$ (20)

where ${\text{h}}_{ij}\in {\mathbb{R}}^{d_{h}}$ is the contextualized token representation.

The sentence-level representation is obtained by attentive pooling:


$$\begin{array}{l} \beta_{ij}=\frac{\exp\left({\text{v}}_{\beta }^{⊤}\tanh\left({\text{W}}_{\beta }{\text{h}}_{ij}\right)\right)}{\sum_{j^{\prime }=1}^{{ñ}_{i}}\exp\left({\text{v}}_{\beta }^{⊤}\tanh\left({\text{W}}_{\beta }{\text{h}}_{ij^{\prime }}\right)\right)}, \end{array}$$ (21)

$$\begin{array}{l} {\text{z}}_{i}=\overset{{ñ}_{i}}{\underset{j=1}{\sum }}\beta_{ij}{\text{h}}_{ij}. \end{array}$$ (22)

The resulting token-level matrix **H**_*i*_ and sentence-level vector **z**_*i*_ serve as the semantic basis for subsequent culture adaptation and skill grounding.

### 3.5 Culture adaptation layer

This module aimed to reduce language- and culture-specific expression bias while preserving educationally meaningful semantic cues. In plain terms, two learners may express the same educational judgment, such as “more practice is needed,” with varying levels of directness, idiomaticity, or emotional intensity. We therefore used a culture-invariant component to represent shared educational meaning and a culture-specific component to represent context-dependent expressive variation. These terms refer to learned representations, not to fixed psychological traits of learners.

#### 3.5.1 Culture-invariant semantic projection

We first project the sentence representation into a shared semantic space:


$$\begin{array}{l} {\text{z}}_{i}^{\text{inv}}=\tanh\left({\text{W}}_{\text{inv}}{\text{z}}_{i}+{\text{b}}_{\text{inv}}\right). \end{array}$$ (23)

#### 3.5.2 Culture-specific mixture-of-experts

To model culture-conditioned expressive patterns, we construct a mixture-of-experts (MoE) layer over the cultural context representation. Let ${\left\{E_{m}\left(\cdot \right)\right\}}_{m=1}^{M_{\text{exp}}}$ denote a set of experts. The routing weights are computed as


$$\begin{array}{l} \pi_{im}=\frac{\exp\left({\text{w}}_{m}^{⊤}{\text{c}}_{i}\right)}{\sum_{m^{\prime }=1}^{M_{\text{exp}}}\exp\left({\text{w}}_{m^{\prime }}^{⊤}{\text{c}}_{i}\right)}. \end{array}$$ (24)

The culture-specific representation is then given by


$$\begin{array}{l} {\text{z}}_{i}^{\text{spec}}=\overset{M_{\text{exp}}}{\underset{m=1}{\sum }}\pi_{im}E_{m}\left({\text{z}}_{i}\right). \end{array}$$ (25)

#### 3.5.3 Culture-aware fusion

We combined the invariant and specific components to obtain the final culture-adapted sentence representation:


$$\begin{array}{l} {\text{z}}_{i}^{\text{cult}}=\text{LayerNorm}\left({\text{W}}_{f}\left[{\text{z}}_{i}^{\text{inv}};{\text{z}}_{i}^{\text{spec}};{\text{c}}_{i}\right]+{\text{b}}_{f}\right). \end{array}$$ (26)

To propagate culture-aware information to the token level, we define


$$\begin{array}{l} {\tilde{\text{h}}}_{ij}=\text{LayerNorm}\left({\text{W}}_{h}\left[{\text{h}}_{ij};{\text{z}}_{i}^{\text{cult}}\right]+{\text{b}}_{h}\right). \end{array}$$ (27)

#### 3.5.4 Adversarial cultural disentanglement

To encourage ${\text{z}}_{i}^{\text{inv}}$ to be less predictive of culture labels, we adopted a gradient reversal layer (GRL) and an adversarial discriminator. Let U denote the set of cultural labels used for adversarial supervision, such as language–platform–domain combinations. The discriminator output is


$$\begin{array}{l} {\hat{\text{u}}}_{i}^{\text{cult}}=\text{softmax}\left({\text{W}}_{d}\text{GRL}\left({\text{z}}_{i}^{\text{inv}}\right)+{\text{b}}_{d}\right). \end{array}$$ (28)

### 3.6 Skill grounding layer

The purpose of this module is to project multilingual educational opinions into an explicit vocational skill cognition space, thereby moving beyond generic satisfaction modeling.

#### 3.6.1 Skill prototype alignment

Given the culture-adapted review representation ${\text{z}}_{i}^{\text{cult}}$ and the external skill prototypes ${\left\{{\text{r}}_{k}^{\text{skill}}\right\}}_{k=1}^{K}$, we compute skill-alignment weights:


$$\begin{array}{l} \alpha_{ik}^{\text{skill}}=\frac{\exp({\left({\text{W}}_{z}{\text{z}}_{i}^{\text{cult}}\right)}^{⊤}\left({\text{W}}_{r}^{\prime }{\text{r}}_{k}^{\text{skill}}\right))}{\sum_{k^{\prime }=1}^{K}\exp({\left({\text{W}}_{z}{\text{z}}_{i}^{\text{cult}}\right)}^{⊤}\left({\text{W}}_{r}^{\prime }{\text{r}}_{k^{\prime }}^{\text{skill}}\right))}. \end{array}$$ (29)

A review-level skill summary is then obtained as


$$\begin{array}{l} {\text{r}}_{i}^{\text{agg}}=\overset{K}{\underset{k=1}{\sum }}\alpha_{ik}^{\text{skill}}{\text{r}}_{k}^{\text{skill}}. \end{array}$$ (30)

#### 3.6.2 Category-specific skill-aware representation

For each skill category *c*^(*k*)^, we construct a category-specific representation:


$$\begin{array}{l} {\text{u}}_{ik}=\text{LayerNorm}\left({\text{W}}_{u}\left[{\text{z}}_{i}^{\text{cult}};{\text{r}}_{k}^{\text{skill}};{\tilde{\text{r}}}_{i}^{\text{meta}};{\text{r}}_{i}^{\text{agg}}\right]+{\text{b}}_{u}\right). \end{array}$$ (31)

Based on **u**_*ik*_, the model predicts whether the *k*-th skill category is activated:


$$\begin{array}{l} {ŷ}_{ik}^{\text{cat}}=\sigma \left({\text{w}}_{\text{cat}}^{⊤}{\text{u}}_{ik}+b_{\text{cat}}\right), \end{array}$$ (32)

where σ(·) is the sigmoid function.

This design allows CA-MuSiC to perform explicit skill-grounded reasoning at the category level rather than treating all educational feedback as undifferentiated sentiment.

### 3.7 Hybrid extractive–generative prediction layer

To handle both explicit span-based sentiment structures and implicit, compositionally complex educational reviews, CA-MuSiC adopts a hybrid extractive–generative prediction strategy.

#### 3.7.1 Extractive prediction head

The extractive head performs token-level sequence labeling for aspect and opinion extraction. For aspect extraction, we define the BIO tag set:


$$\begin{array}{l} T^{a}=\left\{\text{B-A},\text{I-A},\text{O}\right\}. \end{array}$$ (33)

The aspect tagging logits and probabilities are


$$\begin{array}{l} {\text{o}}_{ij}^{a}={\text{W}}_{\text{tag}}^{a}{\tilde{\text{h}}}_{ij}+{\text{b}}_{\text{tag}}^{a}, \end{array}$$ (34)

$$\begin{array}{l} {\text{p}}_{ij}^{a}=\text{softmax}\left({\text{o}}_{ij}^{a}\right). \end{array}$$ (35)

For opinion extraction, we define


$$\begin{array}{l} T^{o}=\left\{\text{B-O},\text{I-O},\text{O}\right\}, \end{array}$$ (36)

with


$$\begin{array}{l} {\text{o}}_{ij}^{o}={\text{W}}_{\text{tag}}^{o}{\tilde{\text{h}}}_{ij}+{\text{b}}_{\text{tag}}^{o}, \end{array}$$ (37)

$$\begin{array}{l} {\text{p}}_{ij}^{o}=\text{softmax}\left({\text{o}}_{ij}^{o}\right). \end{array}$$ (38)

For category-level polarity prediction in the extractive branch, we estimate


$$\begin{array}{l} {\hat{\text{y}}}_{ik}^{\text{pol, ext}}=\text{softmax}\left({\text{W}}_{\text{pol}}^{\text{ext}}{\text{u}}_{ik}+{\text{b}}_{\text{pol}}^{\text{ext}}\right), \end{array}$$ (39)

where ${\hat{\text{y}}}_{ik}^{\text{pol, ext}}\in {\mathbb{R}}^{\left|S\right|}$ is the category-specific polarity distribution.

We also predict sentence-level sentiment from the culture-adapted review representation:


$$\begin{array}{l} {\hat{\text{y}}}_{i}^{\text{sent, ext}}=\text{softmax}\left({\text{W}}_{\text{sent}}^{\text{ext}}{\text{z}}_{i}^{\text{cult}}+{\text{b}}_{\text{sent}}^{\text{ext}}\right). \end{array}$$ (40)

#### 3.7.2 Generative prediction head with mT5 decoder

The generative branch is implemented with an mT5 decoder (Xue et al., 2021). Let **U**_*i*_ = {**u**_*i*1_, …, **u**_*iK*_} denote the set of skill-grounded category representations. We construct an encoder-side memory representation:


$$\begin{array}{l} {\text{M}}_{i}=\left[{\tilde{\text{H}}}_{i};{\text{z}}_{i}^{\text{cult}};{\text{u}}_{i1};\ldots ;{\text{u}}_{iK}\right], \end{array}$$ (41)

where ${\tilde{\text{H}}}_{i}=\left[{\tilde{\text{h}}}_{i1},\ldots ,{\tilde{\text{h}}}_{i{ñ}_{i}}\right]$.

The generative head outputs a structured target sequence


$$\begin{array}{l} {\text{y}}_{i}^{\text{gen}}=\left[y_{i1}^{\text{gen}},y_{i2}^{\text{gen}},\ldots ,y_{iT_{i}}^{\text{gen}}\right], \end{array}$$ (42)

where *T*_*i*_ is the decoding length. At decoding step *t*,


$$\begin{array}{l} {\text{d}}_{it}=\text{mT5Decoder}\left({\text{y}}_{i,<t}^{\text{gen}},{\text{M}}_{i}\right), \end{array}$$ (43)

and the token distribution is


$$\begin{array}{l} P\left(y_{it}^{\text{gen}}|{\text{y}}_{i,<t}^{\text{gen}},{\text{M}}_{i}\right)=\text{softmax}\left({\text{W}}_{\text{gen}}{\text{d}}_{it}+{\text{b}}_{\text{gen}}\right). \end{array}$$ (44)

By constrained decoding and tuple parsing, the generated sequence is converted into category-specific polarity distributions:


$$\begin{array}{l} {\hat{\text{y}}}_{ik}^{\text{pol, gen}}=\text{ParsePol}\left({\text{y}}_{i}^{\text{gen}},c^{\left(k\right)}\right), \end{array}$$ (45)

where ParsePol(·) denotes the deterministic parser that extracts the polarity distribution for category *c*^(*k*)^ from the generated structure.

#### 3.7.3 Hybrid fusion

The final polarity prediction combines the extractive and generative branches:


$$\begin{array}{l} {\hat{\text{y}}}_{ik}^{\text{pol}}=\lambda_{i}{\hat{\text{y}}}_{ik}^{\text{pol, ext}}+\left(1-\lambda_{i}\right){\hat{\text{y}}}_{ik}^{\text{pol, gen}}, \end{array}$$ (46)

where the fusion coefficient is dynamically estimated as


$$\begin{array}{l} \lambda_{i}=\sigma \left({\text{w}}_{\lambda }^{⊤}{\text{z}}_{i}^{\text{cult}}+b_{\lambda }\right). \end{array}$$ (47)

#### 3.7.4 Cross-head consistency modeling

To encourage the two branches to produce coherent predictions, we adopted a symmetric KL-divergence regularization:


$$\begin{array}{l} L_{\text{cons}}^{\left(i,k\right)}=D_{\text{KL}}\left({\hat{\text{y}}}_{ik}^{\text{pol, ext}}∥{\hat{\text{y}}}_{ik}^{\text{pol, gen}}\right)+D_{\text{KL}}\left({\hat{\text{y}}}_{ik}^{\text{pol, gen}}∥{\hat{\text{y}}}_{ik}^{\text{pol, ext}}\right). \end{array}$$ (48)

The overall consistency loss is


$$\begin{array}{l} L_{\text{cons}}=\frac{1}{N}\overset{N}{\underset{i=1}{\sum }}\frac{1}{K}\overset{K}{\underset{k=1}{\sum }}L_{\text{cons}}^{\left(i,k\right)}. \end{array}$$ (49)

### 3.8 Pseudo-label bootstrapping with three-teacher ensemble

Because the target-domain MOOC review corpus lacks large-scale gold annotations for skill-grounded sentiment extraction, CA-MuSiC leverages unlabeled target-domain reviews via a three-teacher ensemble pseudo-label bootstrapping strategy.

Let $D_{T}^{u}={\left\{x_{j}^{u}\right\}}_{j=1}^{N_{u}}$ denote the unlabeled target-domain set. We train three teacher models with different random seeds or checkpoint trajectories:


$$\begin{array}{l} \left\{f_{\theta^{\left(1\right)}},f_{\theta^{\left(2\right)}},f_{\theta^{\left(3\right)}}\right\}. \end{array}$$ (50)

For each unlabeled review $x_{j}^{u}$ and skill category *k*, the ensembled category activation is computed by probability averaging:


$$\begin{array}{l} {ŷ}_{jk}^{\text{cat, ens}}=1\left[\frac{1}{3}\overset{3}{\underset{r=1}{\sum }}{ŷ}_{jk}^{\text{cat},\left(r\right)}\geq \tau_{\text{cat}}\right], \end{array}$$ (51)

where τ_cat_ is the category activation threshold.

For polarity prediction, we aggregate the teacher outputs by majority voting:


$$\begin{array}{l} {ŷ}_{jk}^{\text{pol, ens}}=\text{Vote}\left({ŷ}_{jk}^{\text{pol},\left(1\right)},{ŷ}_{jk}^{\text{pol},\left(2\right)},{ŷ}_{jk}^{\text{pol},\left(3\right)}\right). \end{array}$$ (52)

Let $A_{j}=\left\{k|{ŷ}_{jk}^{\text{cat, ens}}=1\right\}$ denote the set of activated skill categories for review $x_{j}^{u}$. We define the ensemble confidence score as


$$\begin{array}{l} \gamma_{j}=\frac{1}{|A_{j}|+\epsilon }\underset{k\in A_{j}}{\sum }\frac{1}{3}\overset{3}{\underset{r=1}{\sum }}\underset{s\in S}{\max}{ŷ}_{jk,s}^{\text{pol},\left(r\right)}, \end{array}$$ (53)

where ϵ is a small constant for numerical stability.

We further define an agreement score:


$$\begin{array}{l} \eta_{j}=\frac{1}{|A_{j}|+\epsilon }\underset{k\in A_{j}}{\sum }1[{ŷ}_{jk}^{\text{pol},\left(1\right)}={ŷ}_{jk}^{\text{pol},\left(2\right)}={ŷ}_{jk}^{\text{pol},\left(3\right)}]. \end{array}$$ (54)

Only reviews satisfying


$$\begin{array}{l} \gamma_{j}\geq \tau \;\text{and }\eta_{j}\geq \eta \end{array}$$ (55)

are retained as pseudo-labeled samples, where τ and η denote the confidence and agreement thresholds, respectively.

Let ${\tilde{D}}_{T}^{p}$ denote the retained pseudo-labeled set. The pseudo-label supervision loss is then defined as


$$\begin{array}{l} L_{\text{pseudo}}=L_{\text{cat}}^{p}+L_{\text{pol}}^{p}, \end{array}$$ (56)

where $L_{\text{cat}}^{p}$ and $L_{\text{pol}}^{p}$ are computed in the same form as the supervised category activation and polarity losses, but using pseudo labels from the teacher ensemble.

### 3.9 Training objective

To jointly optimize the proposed model, we combine the objectives for extraction, classification, generation, adversarial alignment, skill alignment, and pseudo-label supervision.

#### 3.9.1 Aspect extraction loss

Let ${\text{y}}_{ij}^{a}$ denote the gold BIO label distribution for aspect tagging. The aspect extraction loss is


$$\begin{array}{l} L_{\text{asp}}=-\frac{1}{N}\overset{N}{\underset{i=1}{\sum }}\overset{{ñ}_{i}}{\underset{j=1}{\sum }}\underset{t\in T^{a}}{\sum }y_{ij,t}^{a}\log p_{ij,t}^{a}. \end{array}$$ (57)

#### 3.9.2 Opinion extraction loss

Let ${\text{y}}_{ij}^{o}$ denote the gold BIO label distribution for opinion tagging. The opinion extraction loss is


$$\begin{array}{l} L_{\text{opn}}=-\frac{1}{N}\overset{N}{\underset{i=1}{\sum }}\overset{{ñ}_{i}}{\underset{j=1}{\sum }}\underset{t\in T^{o}}{\sum }y_{ij,t}^{o}\log p_{ij,t}^{o}. \end{array}$$ (58)

#### 3.9.3 Skill category activation loss

The category activation loss is defined as


$$\begin{array}{l} L_{\text{cat}}=-\frac{1}{N}\overset{N}{\underset{i=1}{\sum }}\overset{K}{\underset{k=1}{\sum }}[y_{ik}^{\text{cat}}\log{ŷ}_{ik}^{\text{cat}}+\left(1-y_{ik}^{\text{cat}}\right)\log\left(1-{ŷ}_{ik}^{\text{cat}}\right)]. \end{array}$$ (59)

#### 3.9.4 Category-level polarity loss

Let $y_{ik,s}^{\text{pol}}$ denote the one-hot polarity label for category *k* and sentiment class *s*. The category-level polarity loss is


$$\begin{array}{l} L_{\text{pol}}=-\frac{1}{N}\overset{N}{\underset{i=1}{\sum }}\overset{K}{\underset{k=1}{\sum }}y_{ik}^{\text{cat}}\underset{s\in S}{\sum }y_{ik,s}^{\text{pol}}\log{ŷ}_{ik,s}^{\text{pol}}. \end{array}$$ (60)

#### 3.9.5 Sentence-level sentiment loss

Let $y_{i,s}^{\text{sent}}$ denote the one-hot sentence-level sentiment label. The sentence-level sentiment loss is


$$\begin{array}{l} L_{\text{sent}}=-\frac{1}{N}\overset{N}{\underset{i=1}{\sum }}\underset{s\in S}{\sum }y_{i,s}^{\text{sent}}\log{ŷ}_{i,s}^{\text{sent, ext}}. \end{array}$$ (61)

#### 3.9.6 Generative sequence loss

Let ${\text{y}}_{i}^{\text{gen}*}$ denote the gold target sequence. The generative loss is


$$\begin{array}{l} L_{\text{gen}}=-\frac{1}{N}\overset{N}{\underset{i=1}{\sum }}\overset{T_{i}}{\underset{t=1}{\sum }}\log P\left(y_{it}^{\text{gen}*}|{\text{y}}_{i,<t}^{\text{gen}*},{\text{M}}_{i}\right). \end{array}$$ (62)

#### 3.9.7 Adversarial cultural disentanglement loss

Let $u_{i,c}^{\text{cult}}$ denote the one-hot cultural label for class c∈U. The adversarial loss is


$$\begin{array}{l} L_{\text{adv}}=-\frac{1}{N}\overset{N}{\underset{i=1}{\sum }}\overset{\left|U\right|}{\underset{c=1}{\sum }}u_{i,c}^{\text{cult}}\log{û}_{i,c}^{\text{cult}}. \end{array}$$ (63)

#### 3.9.8 Skill alignment loss

To align the learned category-specific representation with the external skill prototype, we minimize


$$\begin{array}{l} L_{\text{align}}=\frac{1}{N}\overset{N}{\underset{i=1}{\sum }}\overset{K}{\underset{k=1}{\sum }}y_{ik}^{\text{cat}}\left(1-\frac{{\text{u}}_{ik}^{⊤}{\text{r}}_{k}^{\text{skill}}}{||{\text{u}}_{ik}{||}_{2}||{\text{r}}_{k}^{\text{skill}}{||}_{2}}\right). \end{array}$$ (64)

#### 3.9.9 Overall training objective

The overall training objective is defined as


$$\begin{array}{l} L_{\text{total}}=\lambda_{1}L_{\text{asp}}+\lambda_{2}L_{\text{opn}}+\lambda_{3}L_{\text{cat}}+\lambda_{4}L_{\text{pol}}+\lambda_{5}L_{\text{sent}}+\lambda_{6}L_{\text{gen}}\\ \;+\lambda_{7}L_{\text{adv}}+\lambda_{8}L_{\text{align}}+\lambda_{9}L_{\text{cons}}+\lambda_{10}L_{\text{pseudo}}. \end{array}$$ (65)

### 3.10 Multi-stage training procedure

To fully exploit the complementary strengths of the public datasets used in this study, CA-MuSiC is trained in a multi-stage manner rather than through naive one-shot joint training.

#### 3.10.1 Stage 1: multilingual ABSA pre-training

In the first stage, the model is trained on the multilingual ABSA dataset:


$$\begin{array}{l} L^{\left(1\right)}=\lambda_{1}L_{\text{asp}}+\lambda_{2}L_{\text{opn}}+\lambda_{4}L_{\text{pol}}+\lambda_{6}L_{\text{gen}}+\lambda_{7}L_{\text{adv}}. \end{array}$$ (66)

#### 3.10.2 Stage 2: education-domain adaptation

In the second stage, the pre-trained model is adapted to the education-domain dataset:


$$\begin{array}{l} L^{\left(2\right)}=\lambda_{1}L_{\text{asp}}+\lambda_{2}L_{\text{opn}}+\lambda_{3}L_{\text{cat}}+\lambda_{4}L_{\text{pol}}+\lambda_{5}L_{\text{sent}}+\lambda_{6}L_{\text{gen}}\\ \;+\lambda_{7}L_{\text{adv}}+\lambda_{9}L_{\text{cons}}. \end{array}$$ (67)

#### 3.10.3 Stage 3: target-domain skill-grounded adaptation

In the third stage, the model is adapted to the target-domain MOOC reviews:


$$\begin{array}{l} L^{\left(3\right)}=\lambda_{3}L_{\text{cat}}+\lambda_{4}L_{\text{pol}}+\lambda_{5}L_{\text{sent}}+\lambda_{6}L_{\text{gen}}+\lambda_{8}L_{\text{align}}+\lambda_{9}L_{\text{cons}}\\ \;+\lambda_{10}L_{\text{pseudo}}. \end{array}$$ (68)

#### 3.10.4 Algorithmic summary

The full training procedure is summarized in Algorithm 1.

Algorithm 1 Multi-stage training of CA-MuSiC with three-teacher ensemble pseudo labeling. 
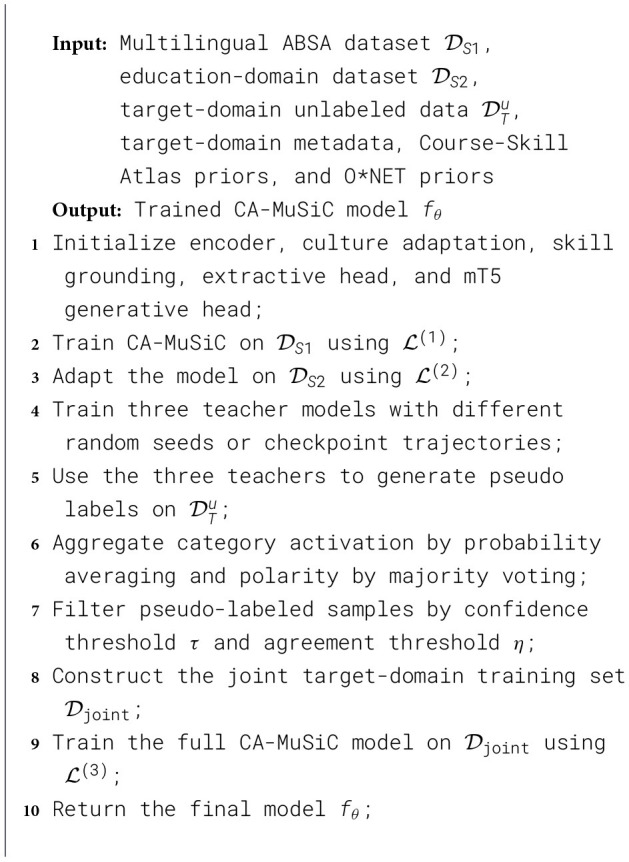

## 4 Experiments

In this section, we comprehensively evaluated the proposed CA-MuSiC model from three complementary perspectives. First, we assessed its ability to perform multilingual aspect-based sentiment reasoning on a general multilingual ABSA benchmark. Second, we examined its fine-grained performance in understanding educational sentiment on an education-domain ABSA benchmark. Third, we evaluated its skill-grounded sentiment generalization on an English–Chinese cross-lingual target-domain MOOC review benchmark constructed entirely from publicly accessible resources. We further conduct systematic ablation studies to quantify the contribution of each major module and training stage.

### 4.1 Experimental goals

The experiments are designed to answer the following research questions:

- RQ1: Does CA-MuSiC outperform recent strong baselines on multilingual ABSA, education-domain ABSA, and target-domain skill-grounded sentiment understanding?
- RQ2: How much do culture adaptation, skill grounding, hybrid extractive–generative prediction, pseudo-label bootstrapping, and consistency regularization contribute to the final performance?
- RQ3: Is the proposed multi-stage training strategy necessary for robust transfer from general ABSA to education-domain and target-domain MOOC review understanding?

### 4.2 Datasets

To ensure reproducibility, all resources used in this study are publicly accesible.

#### 4.2.1 M-ABSA

We use M-ABSA as the multilingual ABSA benchmark (Wu et al., 2025). This dataset spans multiple languages and domains and serves as a suitable benchmark for evaluating multilingual aspect-based sentiment reasoning. In this study, it is used for Main Metric A, namely TASD Micro-F1. We follow the official task definition and benchmark split of the original dataset.

#### 4.2.2 EduRABSA

We use EduRABSA as the education-domain fine-grained sentiment benchmark (Hua et al., 2025). EduRABSA contains tertiary education review texts annotated with aspect–opinion–category–sentiment structures, making it a realistic setting for educational sentiment analysis. It is used for Main Metric B, namely ASQE F1. We follow the official split and annotation scheme provided by the original release.

#### 4.2.3 Target-domain MOOC review benchmark

For target-domain evaluation, we construct a public-data-only English–Chinese cross-lingual MOOC review benchmark based on MOOCCubeX and 100K Coursera Reviews (Yu et al., 2021, 2020). MOOCCubeX provides large-scale MOOC-related resources, including course information, comments, and replies, while the Coursera review dataset provides large-scale learner review texts. Together, these two public corpora provide English–Chinese cross-lingual and cross-platform educational review evidence for target-domain evaluation. We therefore describe the target-domain benchmark as bilingual or cross-lingual, rather than fully multilingual. Their use is further supported by prior studies showing that MOOC review and feedback data are useful for sentiment analysis, pedagogical evaluation, and learner perception mining (Onan, 2021; Chen et al., 2022; Chanaa and eddine El Faddouli, 2022; Wang et al., 2021; Peng and Jiang, 2022; Koufakou, 2024).

To support skill-grounded sentiment modeling, we additionally introduce Course-Skill Atlas and O*NET as external skill knowledge resources (Sabet et al., 2024; Handel, 2016; National Research Council, 2010). Course-Skill Atlas is used to associate course descriptions with labor-market-oriented skill profiles, while O*NET provides a standardized occupational skill taxonomy. Based on these two resources, we construct target-domain skill priors aligned with the six predefined skill cognition categories introduced in Section 3.1.

Notably, the Stage 3 target-domain benchmark comprises two functionally distinct parts. For adaptation, we used a pseudo-labeled training set filtered from the unlabeled MOOC review pool. For model selection and final evaluation, we use strictly human-annotated gold-standard development and test sets. This design allows the model to exploit abundant public unlabeled educational feedback while preserving reliable gold-standard evaluation in the target domain.

#### 4.2.4 Annotation protocol and quality control

To rigorously evaluate the model's cross-lingual transfer and skill-grounding capabilities in the target domain, we constructed human-annotated gold-standard development and test sets from the two public MOOC review corpora described above. Specifically, the gold benchmark was created through stratified sampling from the English 100K Coursera Reviews corpus and the Chinese MOOCCubeX corpus in order to preserve both cross-lingual and cross-platform diversity. The final gold evaluation benchmark contains 5, 000 reviews in total, including 2, 000 development instances and 3, 000 test instances.

The annotation team consisted of three graduate annotators with research backgrounds in Educational Data Mining and bilingual proficiency in Chinese and English. A senior postdoctoral researcher served as the adjudicator for difficult cases. Before formal annotation, the annotators were trained using a task-specific codebook derived from the six predefined skill cognition categories and supplemented by external knowledge from O*NET and the Course-Skill Atlas. A pilot annotation round was first conducted on 200 reviews to refine the annotation criteria and align the team's understanding of implicit skill-related expressions. After the guideline revision, the remaining samples were independently annotated for aspect spans, activated skill cognition categories, and corresponding sentiment polarities.

To assess annotation reliability, we report Krippendorff's α for the three sub-tasks. The agreement scores were 0.79 for aspect boundary extraction, 0.72 for skill category classification, and 0.84 for sentiment polarity classification, indicating substantial to almost perfect agreement. For aspect boundary extraction, agreement was computed using token-level BIO annotations. Disagreements were resolved by majority voting, and cases with three-way disagreement were adjudicated by the senior reviewer. These difficult cases accounted for approximately 4.5% of the annotated samples and primarily involved highly implicit or semantically overlapping categories.

The finalized gold benchmark exhibits a naturally long-tailed distribution of categories. Using the category names defined in Section 3.1, the distribution is as follows: practical utility (31.5%), skill acquisition (28.2%), practice adequacy (16.4%), employability relevance (10.8%), credential value (8.1%), and transferability (5.0%). This distribution reflects the real-world imbalance of learner concerns in English–Chinese target-domain MOOC reviews and helps explain the increased difficulty of target-domain evaluation.

#### 4.2.5 Public-only experimental setting

A notable property of our experimental setup is that all raw corpora and external knowledge resources are public and directly obtainable. The target-domain gold evaluation sets were newly annotated by the authors based exclusively on these public resources, while pseudo-label generation was restricted to the unlabeled training-side pool, without using development or test data. For M-ABSA and EduRABSA, we used the official benchmark splits. For the target-domain MOOC benchmark, Stage 3 training relies on high-confidence pseudo-labeled samples, whereas development and test evaluation rely exclusively on human-annotated gold-standard data.

#### 4.2.6 Direct public access entries and dataset statistics

To strengthen reproducibility, we provide direct public access to entries for all data sources used in this study. Table 2 summarizes the official paper pages, repositories, or database portals, together with the scale and role of each resource in the experimental pipeline.

**Table 2:** Direct public access entries and dataset/resource statistics used in this study.

Resource	Direct public access entry	Public scale/statistics	Role in this study
M-ABSA	ACL Anthology page; official GitHub repository	21 languages; 7 domains; multilingual triplet-level ABSA benchmark	Source-domain multilingual ABSA pre-training; Main Metric A (TASD Micro-F1)
EduRABSA	arXiv paper page; official GitHub repository; Zenodo release	6, 500 English tertiary education reviews (3, 000 course, 3, 000 teaching staff, 500 university)	Education-domain adaptation; Main Metric B (ASQE F1)
MOOCCubeX	official GitHub repository	4, 216 courses; 230, 263 videos; 358, 265 exercises; 637, 572 fine-grained concepts; over 296 million behavioral records from 3, 330, 294 students	Target-domain Chinese MOOC corpus; source of public unlabeled reviews for pseudo-label filtering and human-annotated gold evaluation
100K Coursera Reviews	Kaggle dataset page	100K+ course reviews	Target-domain English MOOC review corpus; source of public unlabeled reviews for pseudo-label filtering and human-annotated gold evaluation
Course-Skill Atlas	Scientific Data paper page; figshare data page	Over 3 million course syllabi from nearly 3, 000 U.S. institutions	External course-to-skill prior construction and skill prototype grounding
O*NET	O*NET Resource Center database page; O*NET OnLine portal	Official occupational knowledge/skills/work-activities database; downloadable files and web services	External occupational skill taxonomy and work-activity grounding

### 4.3 Baselines

We compared CA-MuSiC with six recent and representative baselines spanning general ABSA, instruction-based ABSA, sentiment-enhanced ABSA, cross-lingual ABSA, data-augmented cross-lingual ABSA, and implicit fine-grained sentiment extraction.

- EHG (Lv et al., 2023): an efficient hybrid generation model for ABSA;
- InstructABSA (Scaria et al., 2024): an instruction-learning model for unified ABSA prediction;
- ABSA-ESA (Ouyang et al., 2024): a fine-grained sentiment model based on explicit sparse attention;
- QPEN (Zhao et al., 2024): a cross-lingual ABSA method emphasizing semantic projection and transfer;
- LACA (Šmíd et al., 2025): a recent cross-lingual ABSA model based on LLM-driven augmentation;
- iACOS (Xu et al., 2024): a strong baseline for implicit aspect–opinion–sentiment extraction.

These baselines are selected because they cover the capability dimensions most relevant to our task, including multilingual transfer, fine-grained sentiment reasoning, implicit structure extraction, and target-oriented adaptation.

To ensure fair baseline comparisons, all baselines were evaluated using the same official train/development/test splits for M-ABSA and EduRABSA. For the target-domain benchmark, all methods were evaluated on the same human-annotated development and test sets, and no model was allowed to use test data for pseudo-label generation or hyperparameter selection. When official implementations were available, we followed their recommended settings; otherwise, we used comparable encoder backbones and tuned only the learning rate, batch size, and decoding parameters on the development set. The three reported metrics correspond to different datasets and should therefore be interpreted column-wise rather than averaged across columns.

### 4.4 Evaluation metrics

We use three primary evaluation metrics, each corresponding to a different benchmark and task setting:

- Main Metric A: TASD Micro-F1 on M-ABSA;
- Main Metric B: ASQE F1 on EduRABSA;
- Main Metric C: Skill-grounded Sentiment Macro-F1 on the target-domain MOOC benchmark.

Main Metric C is computed as Macro-F1 on the polarity predictions for the activated skill cognition categories in the target-domain benchmark. Because these three metrics correspond to different tasks and datasets, they should be compared *column-wise only*.

### 4.5 Implementation details

All experiments were conducted on one NVIDIA A100 Tensor Core GPU (NVIDIA Corporation, Santa Clara, CA, USA) with 80GB memory. The software environment consisted of Python 3.10 (Python Software Foundation, Wilmington, DE, USA), PyTorch 2.1.2 (PyTorch Foundation, San Francisco, CA, USA), CUDA 12.1 (NVIDIA Corporation, Santa Clara, CA, USA), and Transformers 4.39 (Hugging Face, Inc., New York, NY, USA). For the multilingual encoding backbone, we used xlm-roberta-large (Meta AI, Menlo Park, CA, USA) (Conneau et al., 2020), while the generative branch was implemented with google/mt5-base (Google Research, Mountain View, CA, USA) as the decoder (Xue et al., 2021). The number of experts in the culture adaptation module was set to 4. The maximum input sequence length was set to 256, and the maximum target generation length was set to 128. The dropout rate was fixed at 0.1 throughout all experiments.

We optimized all models using AdamW (PyTorch, PyTorch Foundation, San Francisco, CA, USA) with a linear learning-rate scheduler. The weight decay was set to 0.01, and the warmup ratio was set to 0.1. The batch size was set to 16 for training and 32 for evaluation. Mixed-precision training (FP16) was enabled to improve computational efficiency. Gradient accumulation was performed for 2 steps, resulting in an effective training batch size of 32. To reduce training variance and improve result stability, we used three random seeds: 42, 52, and 62. Unless otherwise specified, all reported results are presented as mean ± standard deviation across these three runs.

CA-MuSiC was trained in three stages following the curriculum described in Section 3.10. In Stage 1 (multilingual ABSA pre-training), the model was trained for 8 epochs, with the encoder learning rate set to 2 × 10^−5^ and the task-head learning rate set to 1 × 10^−4^. In Stage 2 (education-domain adaptation), the model was further trained for 6 epochs, using an encoder learning rate of 1.5 × 10^−5^ and a task-head learning rate of 8 × 10^−5^. In Stage 3 (target-domain skill-grounded adaptation), the model was trained for 10 epochs, with the encoder learning rate set to 1 × 10^−5^ and the task-head learning rate set to 5 × 10^−5^. For Stage 3, the loss weights were set to λ_align_ = 0.2, λ_cons_ = 0.1, and λ_pseudo_ = 0.5.

For target-domain adaptation, we did not use a manually annotated training split. Instead, pseudo labels were generated exclusively from the unlabeled training-side target-domain pool, without using development or test data. The pseudo-labeling module adopted a three-teacher ensemble constructed from models trained with different random seeds (42, 52, and 62). The overall confidence threshold was set to τ = 0.85, the agreement threshold was set to η = 0.67, and the category activation threshold was set to τ_cat_ = 0.5. From a total of 52, 790 unlabeled target-domain reviews, 18, 432 high-confidence pseudo-labeled instances were retained for Stage 3 training. The development and test sets consisted of 2, 000 and 3, 000 strictly human-annotated gold-standard reviews, respectively, and were not used for pseudo-label generation.

During inference, the generative branch used beam search with a beam size of 4 and a length penalty of 0.8. The extractive branch adopted standard token-wise BIO decoding without CRF.

We also report model complexity and runtime feasibility because CA-MuSiC is more computationally demanding than a single-encoder classifier. Table 3 summarizes the key computational costs. The full hybrid model is best suited for offline or scheduled analysis of MOOC feedback dashboards, while the extractive branch can be used as a lighter online option when immediate inference is required.

**Table 3:** Computational cost and deployment characteristics of CA-MuSiC.

Item	Reported value in our implementation
Trainable parameters	Approximately 1.16B parameters: xlm-roberta-large encoder, mT5-base decoder, and adapter/prediction modules
GPU memory	Approximately 46GB during FP16 training with gradient accumulation; approximately 19GB during full hybrid inference
Training time	Approximately 7.8h for Stage 1, 2.1h for Stage 2, and 5.0h for Stage 3, including pseudo-label generation and final adaptation on one A100 80GB GPU
Inference speed	Approximately 8.2 reviews/s with full hybrid decoding and beam size 4; approximately 34.7 reviews/s when only the extractive branch is used
Platform feasibility	Full hybrid decoding is recommended for periodic dashboard updates; the extractive branch is more feasible for near-real-time platform monitoring

### 4.6 Main results

Table 4 reports the overall comparison results on the three main evaluation settings.

**Table 4:** Main comparison results across the three evaluation settings.

Models	Main Metric A	Main Metric B	Main Metric C
EHG	71.25 ± 0.42	58.42 ± 0.65	56.18 ± 0.82
InstructABSA	72.80 ± 0.35	57.65 ± 0.58	58.34 ± 0.75
ABSA-ESA	68.45 ± 0.55	59.10 ± 0.48	61.25 ± 0.60
QPEN	72.10 ± 0.38	54.28 ± 0.72	55.40 ± 0.85
LACA	70.55 ± 0.45	55.85 ± 0.60	63.72 ± 0.52
iACOS	64.38 ± 0.62	60.15 ± 0.45	52.85 ± 0.95
CA-MuSiC (ours)	**74.62** **±0.28**	**61.45** **±0.35**	**69.88** **±0.42**

Main Metric A is TASD Micro-F1 on M-ABSA, Main Metric B is ASQE F1 on EduRABSA, and Main Metric C is Skill-grounded Sentiment Macro-F1 on the target-domain MOOC benchmark. All results are reported as mean ± standard deviation across three runs with random seeds 42, 52, and 62. Best results are shown in bold.

CA-MuSiC achieves the best performance across all three evaluation settings, demonstrating that the proposed model is effective not only for standard multilingual ABSA but also for education-domain and target-domain MOOC review understanding. On Main Metric A, CA-MuSiC outperforms the strongest baseline InstructABSA by 1.82 points. On Main Metric B, it surpasses the strongest baseline iACOS by 1.30 points. Most importantly, on Main Metric C, CA-MuSiC exceeds the strongest baseline LACA by **6.16** points. These improvements are consistently larger than the corresponding run-to-run variations, indicating that the observed gains are unlikely to be explained by random initialization alone. The particularly large gain on Main Metric C suggests that the combination of culture adaptation, skill grounding, and three-teacher pseudo-label bootstrapping is especially effective for understanding domain-specific MOOC reviews.

**Figure 3:**
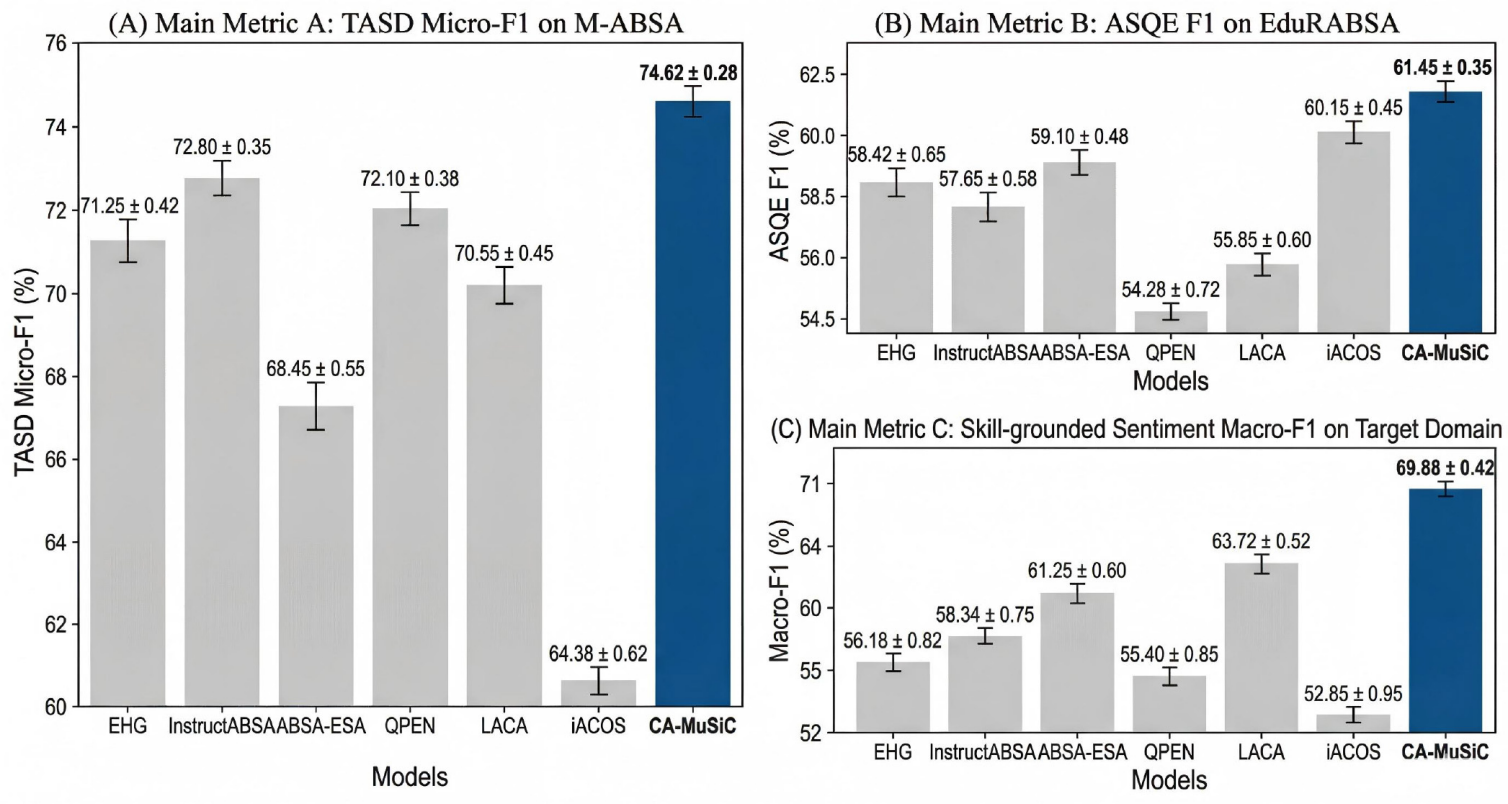
Comparison of CA-MuSiC with strong baselines on the three main evaluation settings. **(A)** reports Main Metric A, namely TASD Micro-F1 on M-ABSA; **(B)** reports Main Metric B, namely ASQE F1 on EduRABSA; and **(C)** reports Main Metric C, namely skill-grounded sentiment Macro-F1 on the target-domain MOOC benchmark. All results are reported as mean ± standard deviation over three runs with random seeds 42, 52, and 62. CA-MuSiC achieves the best performance across all three evaluation settings, with the largest improvement on the target-domain benchmark, highlighting the effectiveness of culture-aware adaptation, skill grounding, and hybrid extractive–generative prediction for understanding multilingual MOOC reviews.

Figure 3 provides a visual comparison of CA-MuSiC and the baseline models on the three evaluation settings. As shown in the figure, CA-MuSiC consistently achieves the best performance across all three metrics, with the largest gain on the target-domain, skill-grounded sentiment task.

### 4.7 Ablation study

To better understand which components of CA-MuTo determine whether SiC is responsible for the observed improvements, we conducted systematic ablation studies. The detailed ablation results for Main Metrics A and B are reported in Table 5, and those for Main Metric C are reported in Table 6.

**Table 5:** Module ablation results on Main Metric A (TASD Micro-F1) and Main Metric B (ASQE F1).

Ablation variants	Metric A	Metric B
CA-MuSiC (full model)	74.62 ± 0.28	61.45 ± 0.35
w/o Culture adapter	71.85 ± 0.65	60.20 ± 0.48
w/o Skill grounding	73.15 ± 0.32	59.85 ± 0.42
w/o Generative head	72.48 ± 0.45	58.92 ± 0.62
w/o Extractive head	72.95 ± 0.40	59.18 ± 0.58
w/o Pseudo-label bootstrapping	73.85 ± 0.35	60.55 ± 0.40
w/o Consistency loss	72.65 ± 0.68	59.35 ± 0.75
Only M-ABSA (S1 only)	73.55 ± 0.38	42.15 ± 1.85
Only EduRABSA (S2 only)	51.28 ± 2.25	60.88 ± 0.42

All results are reported as mean ± standard deviation over three runs.

**Table 6:** Ablation results on Main Metric C (Skill-grounded Sentiment Macro-F1).

Ablation variants	Main Metric C
CA-MuSiC (Full Model)	69.88 ± 0.42
w/o Culture Adapter	67.12 ± 0.75
w/o Skill Grounding	54.25 ± 1.25
w/o Generative Head	68.15 ± 0.55
w/o Extractive Head	68.42 ± 0.48
w/o Pseudo-Label Bootstrapping	59.45 ± 1.45
w/o Consistency Loss	67.95 ± 0.82
Only M-ABSA (S1 only)	48.55 ± 2.10
Only EduRABSA (S2 only)	50.18 ± 1.95

All results are reported as mean ± standard deviation over three runs.

#### 4.7.1 Ablation on Main Metrics A and B

Removing the Culture Adapter leads to a noticeable drop in both metrics, particularly on Main Metric A, suggesting that culture-aware representation learning contributes to multilingual generalization. Removing either the Generative Head or the Extractive Head also degrades performance, especially on Main Metric B, confirming the value of hybrid fine-grained reasoning in educational sentiment analysis. The stage-only variants lead to substantial degradation, especially when the model is trained on a single source stage, indicating that robust performance depends on progressive transfer across multilingual, educational, and target-domain supervision.

#### 4.7.2 Ablation on Main Metric C

**Figure 4:**
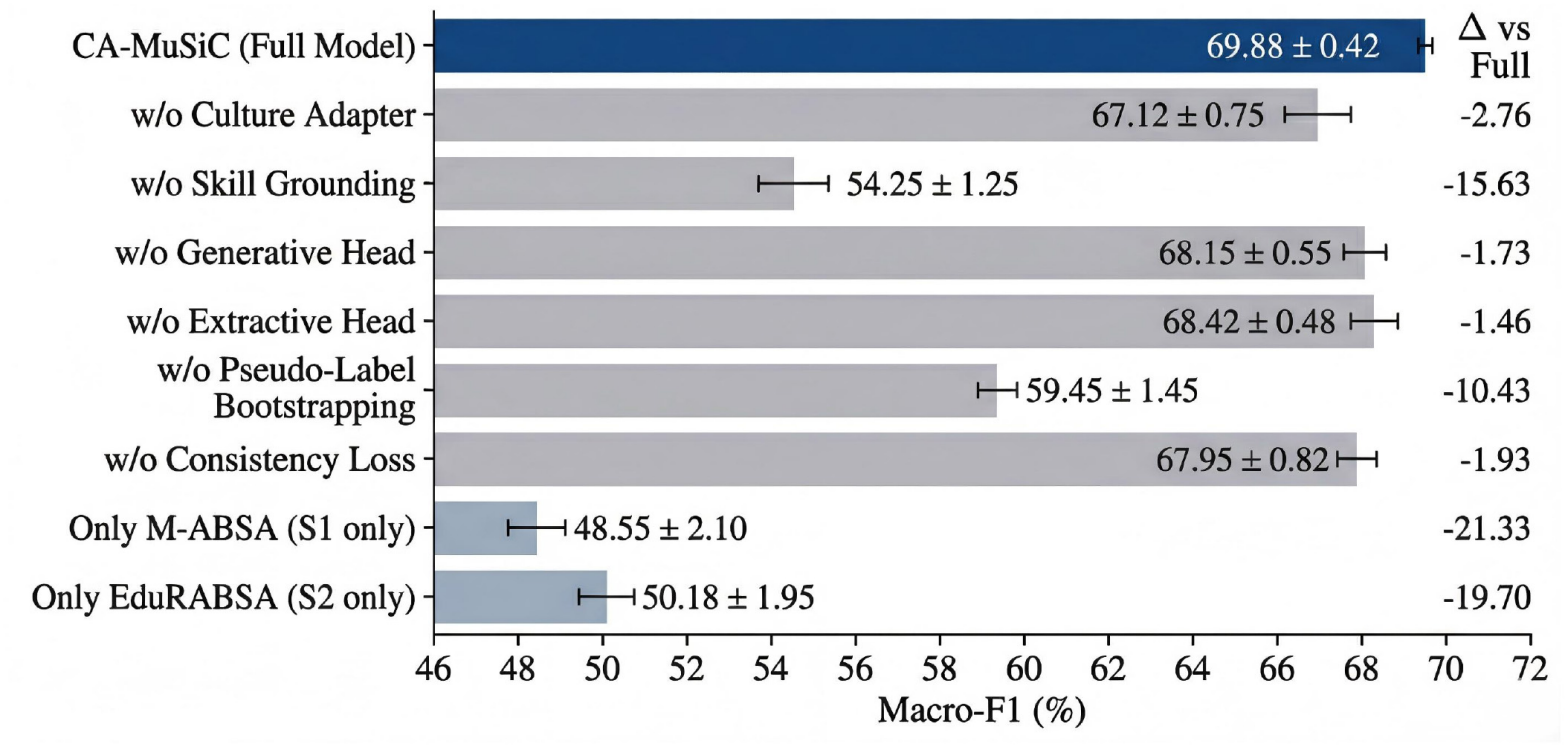
Ablation analysis of CA-MuSiC on the target-domain skill-grounded sentiment task. The figure reports Main Metric C, namely skill-grounded sentiment Macro-F1, on the target-domain MOOC benchmark. Error bars indicate standard deviation over three runs, and the rightmost column shows the performance drop relative to the full model. The results show that skill grounding and pseudo-label bootstrapping are the most critical components, while stage-only training causes the largest degradation in target-domain performance.

Figure 4 visualizes the ablation results on Main Metric C. As shown in the figure, removing skill grounding or pseudo-label bootstrapping causes the most substantial performance degradation, while the stage-only variants lead to the largest overall drops.

Removing Skill Grounding leads to the largest performance drop, which strongly supports the central hypothesis of this paper: target-domain vocational skill cognition cannot be adequately approximated by generic satisfaction or ordinary sentiment classification. Removing Pseudo-Label Bootstrapping also causes a substantial drop and the largest performance fluctuation among all module ablations, indicating that pseudo-label self-training is crucial for both effectiveness and stability under limited target-domain supervision. The usefulness of the Culture Adapter in this target-domain setting further confirms that cross-cultural variation in MOOC review expression is non-negligible.

#### 4.7.3 Parameter analysis

To further evaluate the robustness of the pseudo-labeling strategy, we analyze the sensitivity of the confidence threshold τ used in Stage 3 target-domain adaptation. Table 7 reports the number of retained pseudo-labeled samples, the corresponding retention ratio with respect to the unlabeled target-domain pool, and the target-domain development-set Macro-F1 under different threshold settings. Notably, these results are used for hyperparameter selection on the development set and therefore should not be directly compared with the final test-set Main Metric C reported in the main results.

**Table 7:** Sensitivity analysis of the pseudo-label confidence threshold on the target-domain development set.

Threshold τ	Retained pseudo-labels	Retention ratio	Target-domain Dev Macro-F1 (%)	Interpretation
0.70	~45, 120	85.4%	56.40	Noise overload. Although a large number of samples are retained, many low-confidence or polarity-ambiguous pseudo-labels are introduced, which substantially weaken the model's ability to distinguish negative sentiment and marginal skill cognition signals.
0.80	~28, 650	54.2%	58.75	Performance improves as obviously noisy pseudo-labels are filtered out, allowing the model to learn a more stable approximation to the target-domain distribution.
0.85	18, 432	34.9%	**60.15**	Best trade-off (*sweet spot*). After removing nearly 65% of low-confidence or conflicting pseudo labels, the remaining high-quality samples are sufficient to support effective domain adaptation.
0.90	~8, 240	15.6%	58.10	The threshold becomes overly strict. High-confidence pseudo labels are concentrated on relatively easy samples, reducing the model's exposure to linguistically complex or context-dependent cases.
0.95	~1, 850	3.5%	54.30	Severe degradation. The amount of effective target-domain supervision becomes too small, causing domain adaptation to collapse toward a near-zero-shot setting.

Bold values indicate the best-performing configuration in the sensitivity analysis.

As shown in Table 7, the performance of CA-MuSiC follows a clear non-monotonic pattern as the confidence threshold increases. When τ is too low (e.g., 0.70), the model retains a large number of noisy pseudo labels, which introduces substantial ambiguity into target-domain adaptation and degrades Macro-F1. Increasing the threshold to 0.80 improves performance by removing many unreliable pseudo labels while still preserving sufficient target-domain diversity. The best result is achieved at τ = 0.85, which provides the most effective balance between pseudo-label quality and sample coverage. However, when the threshold becomes overly strict (e.g., 0.90 or 0.95), the retained pseudo-labels are dominated by simpler, more prototypical examples, and the model gradually loses its ability to generalize to harder target-domain cases. These results confirm that pseudo-label filtering should be neither too permissive nor too conservative, and further justify using τ = 0.85 in the final configuration.

### 4.8 Error analysis

To better understand the remaining limitations of CA-MuSiC, we manually inspect representative prediction errors on the target-domain benchmark. Table 8 summarizes four typical failure types, covering implicit skill boundary confusion, culturally specific slang misinterpretation, generative hallucination, and pseudo-label confirmation bias. These cases provide further insight into where the current model still struggles under multilingual and cross-cultural MOOC review settings. As shown in Table 8, the remaining errors of CA-MuSiC mainly arise from four sources. First, the model still struggles with reviews in which vocational skill cognition is expressed only implicitly, without explicit lexical anchors. Second, cross-cultural and colloquial expressions remain challenging when their sentiment orientation depends on localized idioms or discourse conventions. Third, the generative head may occasionally produce semantically appropriate but textually unsupported lexical substitutions, which harms exact-match evaluation. Finally, pseudo-label self-training may introduce category-level confirmation bias, especially when long-tail cognition dimensions are semantically close to more frequent head categories. These observations suggest that future improvements should focus on stronger implicit reasoning, better slang-aware cultural adaptation, hallucination control in generation, and more balanced pseudo-label selection for long-tail categories.

**Table 8:** Representative failure types of CA-MuSiC on the target-domain benchmark.

Error type	Review Example	Gold vs. Prediction	Cause Analysis
Type 1: Implicit Skill Boundary Confusion	“*The certification test was brutal, but worth it for the resume.”* (en)	Gold: [resume, Employability Relevance, Positive] Pred: [certification test, Credential Value, Negative]	No explicit lexical cue directly indicates employability relevance. The course–skill fusion module over-anchors to the salient entity *certification* and fails to infer the implicit job-oriented meaning behind *resume*, leading to both target-span drift and category confusion.
Type 2: Cultural Idiom and Slang Misalignment	*Chinese review (gloss): “The lectures were rather weak, but the final Python project redeemed the course.”*	Gold: [Python final project, Practice Adequacy, Positive] Pred: [lectures, Practical Utility, Negative]	The culture-specific branch and MoE experts do not fully capture localized slang and discourse patterns. Here, the model misses the positive reversal conveyed by a colloquial expression glossed as “*redeem the situation”*, causing polarity routing to fail and the later positive signal to be ignored.
Type 3: Generative Head Hallucination	“*This module on deep learning is exactly what I needed.”* (en)	Gold: [deep learning, Skill Acquisition, Positive] Pred: [neural networks, Skill Acquisition, Positive]	This error arises from the hybrid architecture. During structured generation, the generative head is influenced by pre-trained lexical priors and outputs a semantically similar but textually absent synonym. Although plausible in meaning, the prediction is still incorrect under exact-match evaluation.
Type 4: Pseudo-Label Confirmation Bias	“*The mathematical patterns taught here easily adapt to my physics research.”* (en)	Gold: [mathematical patterns, Transferability, Positive] Pred: [mathematical patterns, Practical Utility, Positive]	*Practical Utility* is a relatively frequent category, whereas *Transferability* is sparser. During iterative pseudo-label self-training, the model becomes biased toward broadly useful positive expressions and tends to collapse cross-domain applicability cases into the majority class.

For layout stability and template compatibility, the Chinese case is shown using an English gloss in the table.

### 4.9 Discussion

The results should be interpreted from both computational and educational-psychological perspectives. Computationally, CA-MuSiC improves over strong ABSA baselines because it does not treat MOOC reviews as generic opinion text. Educationally, the model operationalizes learner reviews as emotionally expressed evaluations of course value, especially perceived skill development. This matters because a negative review may indicate different instructional problems: insufficient practice, weak job relevance, poor transfer to real tasks, or low credential value. These distinctions are important for course redesign and learner-support decisions.

**Figure 5:**
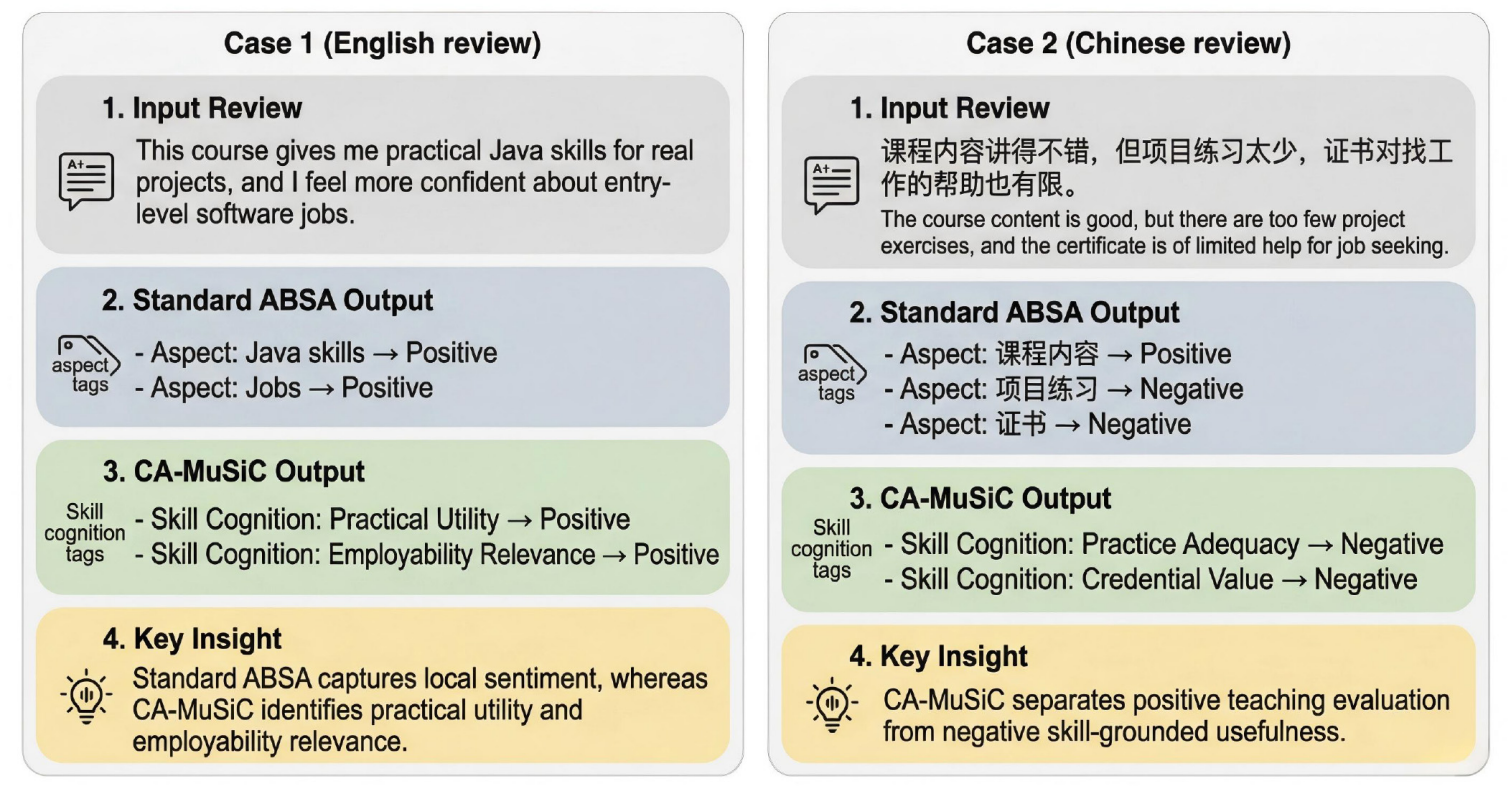
Cross-lingual case studies of skill-grounded sentiment prediction in MOOC reviews. For each example, the figure compares the outputs of standard ABSA and the proposed CA-MuSiC model. The results show that while conventional ABSA can identify local aspect-level sentiment, CA-MuSiC further maps review semantics onto vocational skill cognition dimensions, including practical utility, employability relevance, practice adequacy, and credential value. These examples highlight the value of skill-grounded sentiment modeling for interpreting English–Chinese MOOC reviews under different cultural-context conditions.

Figure 5 illustrates this difference qualitatively. Standard ABSA can identify local positive or negative aspects, but it does not explain which skill-related value is being evaluated. Conversely, for CA-MuSiC, maps review semantics to categories such as practical utility, employability relevance, practice adequacy, and credential value. This mapping makes the output more useful for educational interpretation by linking learners' emotions to a specific dimension of perceived learning value.

The findings also strengthen the educational psychology contribution of the study. First, the model treats learner emotion as evaluative information rather than noise. Emotional polarity is interpreted together with the skill dimension being evaluated. Second, the model supports learner evaluation analysis by distinguishing satisfaction with course content from judgments about usefulness, practice, transfer, and credentials. Third, the model contributes to skill-perception research by showing that perceived skill acquisition and perceived employability value can be modeled from naturally occurring learner-generated feedback. These contributions connect computational review analysis to learner emotion, learner evaluation, and vocational skill cognition in online learning environments.

The cultural-context results should be interpreted cautiously. The culture adaptation module is motivated by cross-cultural psychology, but the empirical metadata are language, platform, and discipline rather than direct measures of cultural values or identity. Thus, the results show that modeling observable cultural-context variation improves cross-lingual review interpretation; they do not prove that the model has captured culture in a complete anthropological or psychological sense. This distinction is important for avoiding overgeneralization or stereotyping. In practical use, CA-MuSiC should be used to identify possible differences in evaluative expression, not to assign cultural traits to individual learners.

The pseudo-labeling results also require careful interpretation. The ablation study shows that pseudo-label bootstrapping substantially improves target-domain performance, but it can also amplify teacher errors. Such bias may be especially harmful for underrepresented languages, platforms, or long-tail categories. For example, the error analysis shows that transferability can be confused with practical utility because the latter is more common. Therefore, improvements from pseudo-labeling should be interpreted as evidence that unlabeled reviews aid target-domain adaptation, not as evidence that the pseudo-labels are free of psychological or cultural bias.

Finally, the model's complexity limits immediate deployment. The full hybrid model provides the best interpretability and performance but requires substantial GPU memory and offline decoding time. For real MOOC platforms, a feasible deployment strategy is to run the full model periodically for dashboard-level feedback analysis, while using the extractive branch for faster monitoring. This distinction affects how the results should be translated into practice: CA-MuSiC is currently more suitable for institutional analytics and research use than for low-resource real-time classroom applications.

## 5 Conclusions

This study proposed CA-MuSiC, a Culture-Aware Multilingual Skill Cognition Model for multilingual MOOC review understanding. By integrating culture-aware representation adaptation, external skill grounding from Course-Skill Atlas and O*NET, a hybrid extractive–generative architecture, and a three-teacher ensemble pseudo-label bootstrapping strategy, CA-MuSiC was designed to address the limitations of conventional sentiment models in cross-cultural educational settings (Sabet et al., 2024; Handel, 2016; National Research Council, 2010; Xue et al., 2021; Šmíd et al., 2025). Experimental results on M-ABSA, EduRABSA, and the target-domain MOOC benchmark showed that the proposed model consistently outperformed strong baselines across all three evaluation settings. Most notably, the largest improvement was observed on the target-domain skill-grounded sentiment task, indicating that vocational skill cognition cannot be adequately reduced to generic course satisfaction or ordinary sentiment classification. The ablation study further demonstrated that skill grounding and pseudo-label bootstrapping are the most critical components for target-domain performance, whereas culture adaptation, hybrid prediction, and consistency regularization also make stable contributions.

At the same time, the present study has several limitations that directly affect interpretation. First, culture is operationalized through metadata on language, platform, and discipline. These proxies help model patterns of contextual expression, but they do not measure individual cultural identity or cultural values. Therefore, the findings should be interpreted as culturally context-aware evidence rather than as a comprehensive cultural explanation. Second, the target-domain benchmark is English–Chinese cross-lingual rather than fully multilingual. The broader model is trained and evaluated with multilingual ABSA resources, but target-domain conclusions should not be generalized to all languages or MOOC cultures without further validation. Third, the predefined six-category skill cognition space improves interpretability but may underrepresent emerging or context-specific learner concerns. Because categories are multi-label and sometimes semantically close, some errors may reflect boundary ambiguity rather than purely technical failure. Fourth, although three-teacher pseudo-labeling improves target-domain adaptation, it may still propagate majority-language, majority-platform, or head-category bias when high-confidence predictions are systematically wrong. Fifth, the full hybrid model is computationally expensive, which limits immediate real-time use on resource-constrained MOOC platforms.

These limitations also point to several promising directions for future research. Future work may extend CA-MuSiC to more languages, educational platforms, and cultural contexts; collect direct survey or interview evidence on learners' skill perceptions; explore hierarchical or dynamic skill taxonomies; and develop uncertainty-aware or human-in-the-loop pseudo-labeling for long-tail categories. It would also be valuable to integrate multimodal educational signals, such as discussion interactions, videos, and learner behavior trajectories, to better understand how culture, emotion, and perceived skill value interact in online higher education.

Overall, this work provides a new computational perspective on understanding MOOC reviews by framing it as a culture-aware, skill-grounded educational interpretation task. By bridging cross-lingual sentiment analysis, educational psychology, learning analytics, and vocational skill knowledge grounding, CA-MuSiC offers both methodological value and practical relevance for future research on interpretable educational intelligence in multilingual learning environments.

## Data Availability

The original contributions presented in the study are included in the article/supplementary material, further inquiries can be directed to the corresponding authors.
